# Microbiota transplantation for cotton leaf curl disease suppression—core microbiome and transcriptome dynamics

**DOI:** 10.1038/s42003-025-07812-7

**Published:** 2025-03-06

**Authors:** Ayesha Badar, Rhea Aqueel, Ali Nawaz, Umer Zeeshan Ijaz, Kauser Abdulla Malik

**Affiliations:** 1 Kauser Abdulla Malik School of Life Sciences, Forman Christian College (A Chartered University), Lahore, Pakistan; 2 Mazumdar-Shaw Advanced Research Centre, Water & Environment Research Group, University of Glasgow, Glasgow, UK; 3 Bioinformatics Group - Department of Digital Health Sciences and Biomedicine, University of Siegen, Siegen, Germany; 4 National University of Ireland, Galway, Ireland; 5 Department of Molecular and Clinical Cancer Medicine, University of Liverpool, Liverpool, UK; 6 Pakistan Academy of Sciences, Islamabad, Pakistan

**Keywords:** Applied microbiology, Microbiome

## Abstract

Microbiota transplantation is a strong tool for managing plant disease. This study investigates the effects of microbiota transplantation on Cotton Leaf Curl Disease (CLCuD) resistance in *Gossypium hirsutum*, a species with good fiber length but high susceptibility to biotic stresses. Using metabarcoding for V3-V4 *16S rRNA* gene amplicon, microbial fractions from both rhizosphere and phyllosphere of CLCuD-resistant species *Gossypium arboreum*, and susceptible cotton varieties are analyzed. Unique bacterial taxa have been identified associated with disease resistance. Interspecies and intraspecies microbiota transplantation is conducted, followed by CLCuD incidence assays. It is seen that rhizospheric microbiota transplantation from *G. arboreum* FDH228 significantly suppresses CLCuD in *G. hirsutum* varieties, outperforming exogenous salicylic acid application. While phyllospheric transplants also reduce disease incidence, they are less effective than rhizospheric transplants. Differential expression analysis DESeq2 is utilized to identify key bacterial genera correlated with CLCuD suppression, including *Pseudoxanthomonas* and *Stenotrophomonas* in the rhizosphere of *G. arboreum* FDH228. Functional pathway analysis reveals upregulation of stress response and metabolism in tolerant species. Transcriptomics reveals upregulation of genes involved in protein phosphorylation and stress response in interspecies rhizospheric microbiota transplants. This study highlights microbiota transplantation as a sustainable method for controlling CLCuD along with specific microbial and genetic mechanisms contributing to CLCuD resistance.

## Abstract (web summary)

Microbial community analysis of rhizospheric and phyllospheric microbiota transplantation to induce Cotton Leaf Curl Disease tolerance in *Gossypium hirsutum*, with transcriptomic examination post-transplantation from *Gossypium arboreum*.

## Introduction

Microbiota transplantation, particularly rhizospheric microbiota transplantation, represents an increasingly advancing approach in plant sciences. It is aimed at enhancing crop health and productivity. The rhizosphere, the narrow region of soil influenced by root secretions and associated soil microorganisms, is crucial for plant growth and nutrient uptake^1^. The bioinoculant market utilizes microbial agents specifically isolated from the plant rhizosphere and offers products to enhance agricultural production, often using single microbial species to promote crop growth or antagonize soil pathogens^2^. However, single inoculations may be prone to challenges provided by certain environmental conditions, leading to insufficient colonization and effectiveness. Selected salicylic acid (SA) producing bacterial isolates have been utilized in mono association as well as a synthetic consortium, to enhance effectiveness and resilience to disease, as reported in our previous study Aqueel et al.^3^.

An improvement over the application of selecting a few microbial species is the complete microbiota transplantation, which involves transferring entire rhizospheric or phyllospheric microbiomes containing millions of beneficial microbes from various species to target crops. This approach supports more diverse and functional microbial communities, promoting plant growth and health^4^. Microbiota transplantation offers superior benefits by leveraging the complexity and functionality of entire microbial communities. It enhances nutrient provision, with microorganisms like mycorrhizal fungi supplying up to 80% of phosphorus and nitrogen to plants. It also supports soil health by fostering a balanced ecosystem, improves disease suppression through broader pathogen resistance, and increases resilience by enhancing adaptability to environmental changes^5^. Thus, microbiota transplantation provides more robust and sustainable agricultural solutions compared to single-cell applications and microbial consortia containing a limited number of bacterial strains with only selected properties^6^.

Microbiota transplantation, specifically rhizospheric microbiota transplantation, has been employed previously by various researchers to control certain microbial diseases in plants. This strategy has been used to tackle fungal pathogens in susceptible plant species to suppress disease intensity^7^. In the current study, we have advanced our research to tackle the viral cotton leaf curl disease (CLCuD) through the transplantation of the entire microbiome, an approach easier and quicker than culturing and designing specific synthetic microbial communities. Cotton leaf curl virus (CLCuV) is a major viral pathogen that affects the cotton crop globally, particularly in South Asia, including Pakistan. The devastation caused by the CLCuV in Pakistan is given in Supplementary Note 1. CLCuV is transmitted by the whitefly (*Bemisia tabaci*), causing significant yield losses by disrupting key physiological processes in plants. Infected plants exhibit symptoms such as vein thickening, leaf curling, enation, and severe stunting, which collectively reduce cotton fiber quality and productivity^8^. These symptoms result from the interaction between viral replication-associated proteins and host defense pathways, leading to host susceptibility^9,10^. In Pakistan, CLCuV has emerged as one of the most devastating diseases for cotton, threatening the viability of high-yielding cotton species like *Gossypium hirsutum*^11^. The country hosts two major cotton species. *Gossypium arboreum (desi cotton)* is a species naturally tolerant to most of the biotic and abiotic stresses but useless for good fiber production owing to a short fiber length (Supplementary Note 1). *Gossypium hirsutum*, on the other hand, is a species with fairly long fiber length, but extremely high susceptibility towards biotic stresses^12^. This makes interspecies microbiota transplantation a promising strategy for restoring the industrial viability of *G. hirsutum*.

Therefore, the primary aim of this study is to see if the same strategy can be employed to suppress a viral pathogen. Furthermore, the use of phyllospheric microbiota transplantation to reduce a lethal viral disease remains unexplored. The phyllosphere is known to be a dynamic habitat for diverse microbiota, and plays a crucial role in plant, microorganism, and atmospheric communication. Studying the dynamics of the phyllospheric microbiota transplantation can advance sustainable agriculture by suggesting mechanisms involved in inducing disease resistance or plant growth promotion. Additionally, it can improve stress-resistance mechanisms to combat biotic and abiotic stresses^13^. Therefore, the secondary aim of this study is to see if the incorporation of phyllospheric microbiota transplantation offers disease-suppressing capabilities.

## Results

### Bacterial diversity analysis of microbial fractions

**Fig. 1:**
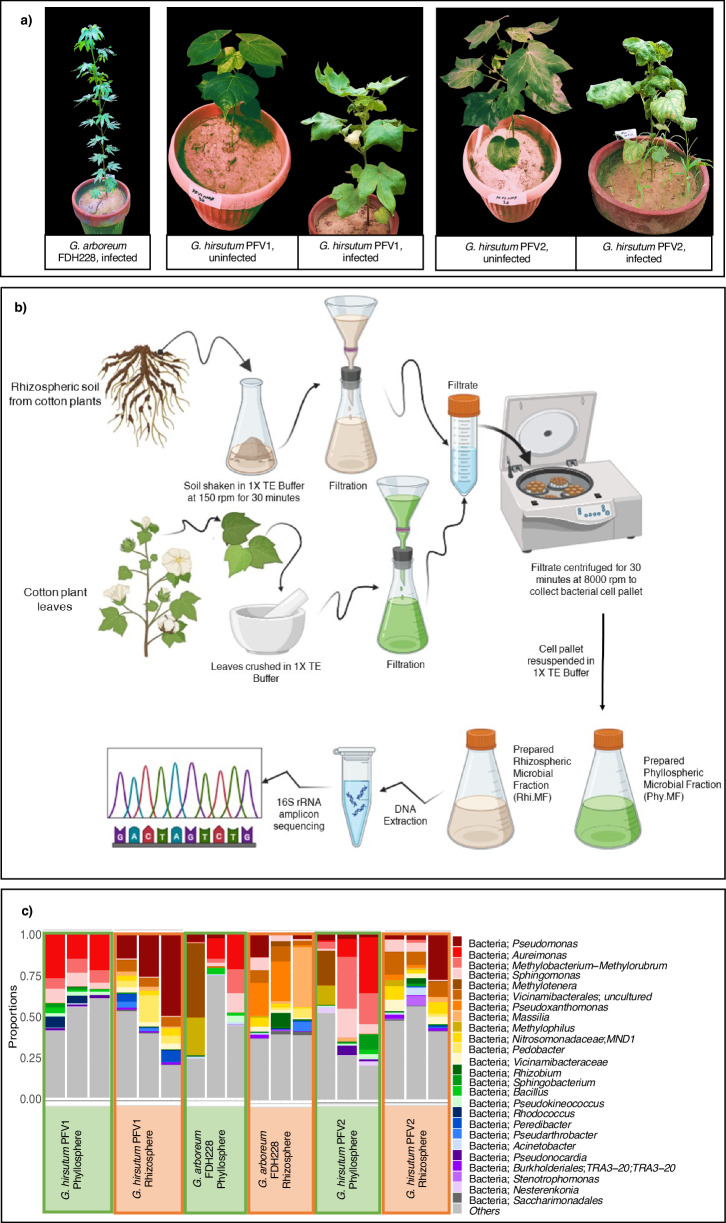
Experimental breakdown for microbial fraction preparation and analysis. **a** Cotton plants grown in an uncontrolled environment till their flowering stage, displaying typical symptoms of CLCuD; *Gossypium arboreum* (FDH 228), completely tolerant to CLCuV, shows no symptoms of leaf curling or stunted growth at extreme viruliferous whitefly attack; *Gossypium hirsutum* (PFV1), partially tolerant to CLCuV, shows limited symptoms of leaf curling and stunted growth as compared to; *Gossypium hirsutum* (PFV2), completely susceptible to CLCuV, shows severe symptoms of leaf curling and stunted growth. **b** The schematic figure was created with Biorender.com and shows the general workflow of the experimentation of the study; extraction of rhizospheric and phyllospheric microbiomes and preparation of microbial fractions (MFs) for microbiota transplantation, DNA isolation from MF samples and analysis of the sequence using Illumina MiSeq Platform. **c** Comparative bacterial diversity of Rhi.MFs and Phy.MFs, Relative abundance to genus level for *G. hirsutum* PFV1 Rhi.MF and Phy.MF, *G. arboreum* FDH228 Rhi.MF and Phy.MF and *G. hirsutum* PFV2 Rhi.MF and Phy.MF.

As concluded in our previous research, bacterial diversity in the cotton plant microbiome is associated with relative resistance levels to CLCuV^3^. While distinct variations can be seen phenotypically in both the CLCuV-infected and uninfected *G. hirsutum* plants (Fig. 1a), the microbial fractions are extracted from both rhizosphere and phyllosphere (Fig. 1b) for analysis of the bacterial diversity based on metabarcoding of V3-V4 *16S rRNA* gene amplicon using Illumina^®^ MiSeq.

The common bacterial genera identified in all the rhizospheric MF samples included *Pseudomonas*, uncultured species of *Vicinamibacterales, Nitrosomonadaceae MND1*, and *Pedobacter* (Fig. 1c). The rhizospheric MF of FDH228 (CLCuD resistant) can be seen to be the most distinct, with several genera like *Pseudoxanthomonas, Massilia* and *Rhizobium* present in much higher abundance than in the rhizospheric MFs of PFV1 and PFV2. Genera like *Peredibacter* and *Pseudarthrobacter* can be seen to be present in FDH228 and PFV1 rhizosphere and not in the rhizosphere of PFV2. *Stenotrophomonas*, on the other hand, is seen to be much higher in abundance in PFV2 rhizospheric MF than in any other MF.

**Fig. 2:**
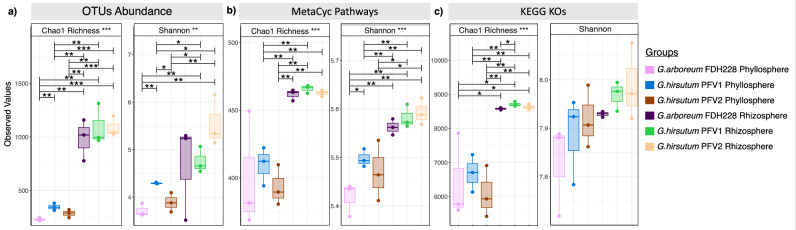
Comparative alpha diversity measures using Chao1 richness and Shannon Diversity. **a** OTUs abundance. **b** MetaCyc pathways. **c** Kegg Orthologs. The lines connect groups according to simple ANOVA with significance values drawn on top as: *p  <  0.05, **p  <  0.01, or ***p  <  0.001.

Similarly, the phyllospheric MF of FDH228 (CLCuD-resistant) *G. arboreum* contains genera like *Methylotenera* and *Methylophilus* in a much higher abundance than in phyllospheric MFs of CLCuD-susceptible *G. hirsutum*, PFV1, and PFV2. The common bacterial genera identified in all the phyllospheric MF samples included *Pseudomonas*, *Aureimonas*, *Methylobacterium*−*Methylorubrum*, and *Sphingomonas*. Figure 2 illustrates the α-diversity analysis of rhizospheric and phyllospheric MF samples from all three varieties of cotton. For PFV1, the OTU abundance (Fig. 2a) was found to be significantly higher in rhizospheric MF than in its phyllospheric counterpart. Similarly, the relative OTU abundance was seen to be significantly higher in rhizospheric MF than the phyllospheric MF both in FDH228 and PFV2.

The culture-based quantification and analysis of microbial diversity in each microbial fraction are given in Supplementary Result 1.

### Elucidating the influence of interspecies microbiota transplantation on CLCuD progress

**Fig. 3:**
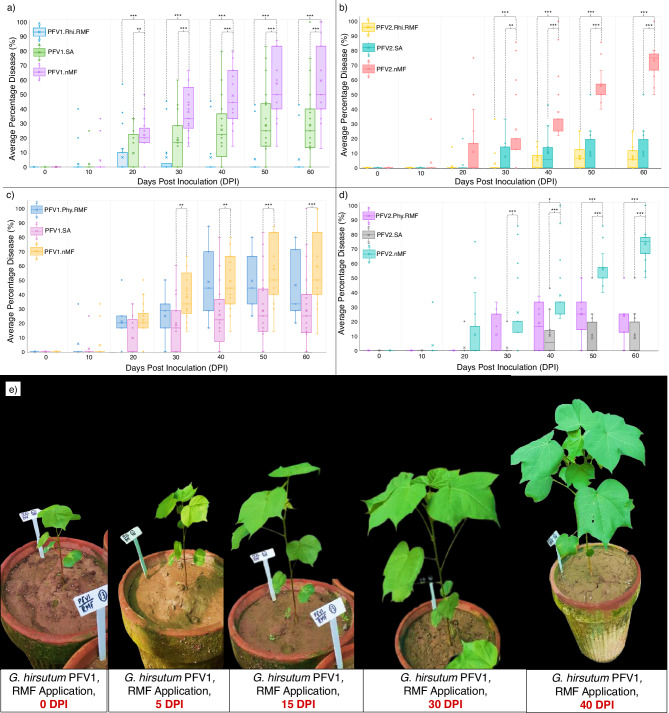
CLCuD progress in cotton varieties treated with rhizospheric microbial fractions (Rhi.MFs), phyllospheric microbial fractions (Phy.MFs), and exogenous salicylic acid. **a** CLCuD progress in *G. hirsutum* partially tolerant variety, PFV1, under Rhi.MFs application. **b** CLCuD progress in *G. hirsutum* completely susceptible variety, PFV2, under Rhi.MFs application. **c** CLCuD progress in *G. hirsutum* partially tolerant variety, PFV1, under Phy.MF application. **d** CLCuD progress in *G. hirsutum* completely susceptible variety, PFV2, under Phy.MFs application. **e** *G. hirsutum*, PFV1 plant under Rhi.RMF application, showing no symptoms of CLCuD until 40 days post-inoculation. The lines connect groups according to Tukey post hoc test with significance values as: *p  <  0.05, **p  <  0.01, or ***p  <  0.001. Each group comprises 30 independent biological replicates.

Since the diversity profiles of rhizospheric and phyllospheric microbiota differ, the microbiota were transplanted separately in both interspecies and intraspecies combinations given in Supplementary Fig. 1. The plants remained under surveillance for 60 days post-viral inoculation and were assessed for CLCuD symptoms every day. Visible differences in CLCuD progress were noticed in groups of *G. hirsutum* PFV1 and PFV2 to which Rhi.RMF was applied. The Average Percentage Disease (APD) was calculated for 30 plants in each group and plotted against 60 days post inoculation (DPI), as illustrated in Fig. 3. The *G. hirsutum* PFV1 group (PFV1.Rhi.RMF) transplanted with *G. arboreum* FDH228 rhizospheric microbiome, outperformed the exogenous SA application group and control group in CLCuD suppression (Fig. 3a). Similarly, rhizospheric microbiota transplantation with the rhizospheric microbiota from *G. arboreum* FDH228 also significantly suppressed CLCuD in the completely susceptible *G. hirsutum* variety, PFV2 (Fig. 3b). Although phyllospheric MF transplantation reduced CLCuD significantly in both the partially tolerant and completely susceptible varieties PFV1 and PFV2, it did not outperform the group exogenously foliar sprayed with SA (Fig. 3c, d). The suppression in CLCuD recorded through disease incidence assays is depicted in Fig. 3e for the plant group PFV1.Rhi.RMF, in a time-lapse photograph from 0 DPI to 40 DPI. The disease severity assays for the remaining combinations of rhizospheric and phyllospheric transplantations are given in Supplementary Fig. 2. The influence of interspecies microbiota transplantation on plant growth is given in Supplementary Result 2.

### DESeq2: key differentiation in bacterial genera in CLCuD-resistant *G. arboreum* and variably susceptible *G. hirsutum*

**Fig. 4:**
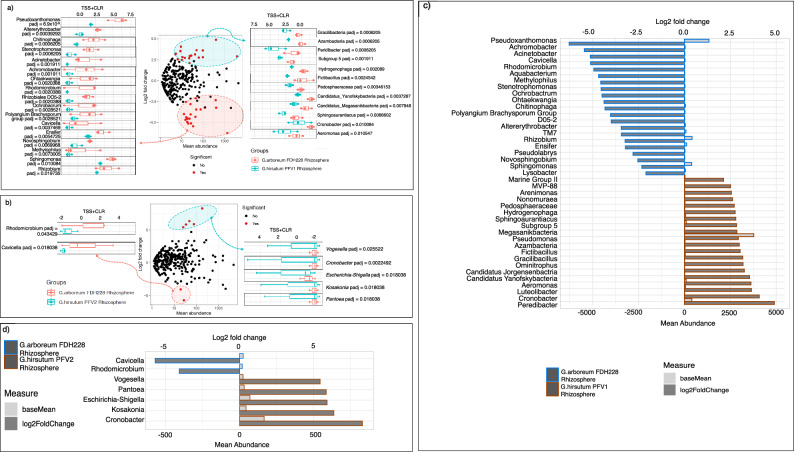
Comparative analysis of Rhi.MFs from *G. arboreum* FDH228 and *G. hirsutum* PFV1 and PFV2 using DESeq2. **a** Log2Fold change in bacterial genera plotted against mean abundance comparing FDH228 rhizospheric MF and PFV1 rhizospheric MF, and key genera with significantly higher abundance in FDH228 rhizospheric MF than in PFV1 rhizospheric MF. **b** Log2Fold change in bacterial genera plotted against mean abundance comparing FDH228 rhizospheric MF and PFV2 rhizospheric MF, and key genera with significantly higher abundance in FDH228 rhizospheric MF than in PFV2 rhizospheric MF. **c** Key selected bacterial genera in FDH228 and PFV1 rhizosphere. **d** Key selected bacterial genera in FDH228 and PFV2 rhizosphere.

Comparative microbial diversity analysis of the CLCuD-resistant *G. arboreum* and partially tolerant *G. hirsutum* PFV1 rhizospheric MF samples performed using DESeq2 is illustrated in Fig. 4a. The key genera found in significantly higher abundance in the rhizospheric MF of FDH228 include *Pseudoxanthomonas, Altererythrobacter, Stenotrophomonas, Achromobacter, Rhodomicrobium,* and *Rhizobiales*, etc, whereas the ones found in significantly higher abundance in the rhizospheric MF of *G. hirsutum* PFV1 include *Gracilibacteria, Azambacteria, Peridibacter, Hydrogenophaga, Fictibacillus,* and *Pedosphaeraceae,* etc. Comparatively, the key genera found to be highly expressed in FDH228 rhizospheric MF as compared to the PFV2 rhizospheric MF include *Rhodomicrobium* and *Cavicella* (Fig. 4b), whereas key genera expressing significantly higher in PFV2 rhizosphere include *Cronobacter, Vogesella, Escherichia-Shigella, Kosakonia,* and *Pantoea*. The DESeq2 comparative analysis of the phyllospheric MF samples is given in Supplementary Figs. 3 and 4. The remaining results for DESeq2 are given in Supplementary Result 3.

### Pathway analysis: basis of plant-microbe interactions and possible disease suppression

The functional potential of the microbial community residing on and inside the plant surfaces was analyzed by employing pathway identification and analysis (Supplementary Figs. 5–8). PICRUSt2 algorithm was, therefore, deployed to identify the MetaCyc pathways. The comparative analysis of MetaCyc pathways differentially expressed in the rhizosphere of CLCuD-resistant *G. arboreum* FDH228 and partially tolerant *G. hirsutum* PFV1 revealed a total of 13 pathways (Supplementary Fig. 5a). Log2fold change plotted against mean abundance shows pathways including peptidoglycan biosynthesis, starch degradation and hydroxyacetophenone degradation to be upregulated in FDH228 rhizosphere. Whereas, PFV1 had upregulated pathways including glycol metabolism and degradation, super pathway of L-arginine, putrescine, and 4-aminobutanoate degradation, super pathway of L-arginine and L-ornithine degradation, naphthalene degradation (aerobic), L-tryptophan biosynthesis, phosphopantothenate biosynthesis III (archaea), methanogenesis, naphthalene degradation to acetyl-CoA and 7-(3-amino-3-carboxypropyl)-wyosine biosynthesis. The super pathway for L-tryptophan biosynthesis was also seen to be enhanced in the PFV2 rhizosphere (Supplementary Fig. 5b). Remaining pathway analyses are given in Supplementary Result 4.

### Core microbiome signatures associated with CLCuD suppression in cotton microbiome transplants

**Fig. 5:**
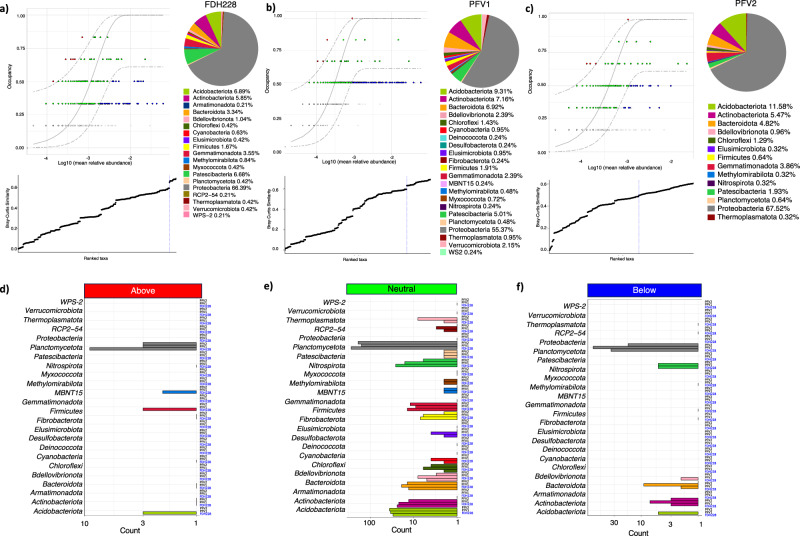
Core microbiome inference and fitting neutral model. Core microbiome [red, green, and blue points in left figure of (**a**) FDH228, (**b**) PFV1, and (**c**) PFV2] identified through species occupancy abundance diagrams incorporating a site-specific occupancy criterion (occupancy being: *phyllosphere*; and *rhizosphere*). To identify the threshold for core microbiome, we calculate the function C (bottom plot of (**a**–**c**), which implicitly incorporatesthe explanatory power of the chosen core subset in terms of capturing beta diversity). The blue dotted line represents the threshold for “Last 2% decrease” criteria where OTUs are incorporated in the core subset until there is no more than 2% decrease in beta diversity. Independently, a neutral model is fitted with those OTUs that fall within the 95% interval confidence intervals shown in green, whilst non-neutral OTUs with observed frequency above the predicted frequency from the neutral model (selected by the host) are shown in red colors, and those with observed frequency below the predicted frequency from the neutral model (selected by dispersal limitation) are shown in green colors. The proportion of core OTUs belonging to different taxonomic levels are shown with pie charts whilst the count of neutral/non-neutral OTUs belonging to different taxonomic levels is shown with the bar plots in **d** Above, **e** Neutral, and **f** Below.

Since the core microbiome of a host organism is the set of microbial genera that it retains despite environmental changes and biotic and abiotic stress, a dive into the specific core microbiomes of each variety of cotton is crucial. Figure 5 shows the core microbiome profiles of the completely tolerant *G. arboreum* FDH228, and partially and completely susceptible *G. hirsutum* PFV1 and PFV2, depicting the variations associated with their relative CLCuD susceptibility levels. FDH228, the completely and naturally resistant *G. arboreum* variety, shows the most diverse core microbiome as depicted by the blue dotted line in the lower graph in Fig. 5a, followed by PFV1, the partially tolerant *G. hirsutum* variety (Fig. 5b), and PFV2, the completely susceptible *G, hirsutum* variety (Fig. 5c). The representation of core genera in Fig. 5a–c further demonstrate the OTUs in each variety’s core microbiome that have been observed to be above the predicted frequency, allotted with red dots, and below the predicted frequency, allotted with blue dots. The taxonomic association of these selected phyla by each variety is shown in Fig. 5d–f. The completely tolerant *G. arboreum* can be seen to have selected more phyla as compared to *G. hirsutum*, namely *Planctomycetota, MBNT15, Firmicutes*, and *Acidobacteriota*. The key phyla downregulated by *G. arboreum* include *Actinobacteriota, Bacteroidota, Bdellovibrionota,* and *Nitrospirota*. The differential taxonomic coverage of core microbiome in the relative rhizospheric and phyllospheric fractions of the three varieties is elucidated in the form of heat trees in Supplementary Fig. 9. The remaining results for core microbiome analyses are given in Supplementary Result 5.

### Beneficial bacterial genera are recruited in the core microbiome for disease suppression

**Fig. 6:**
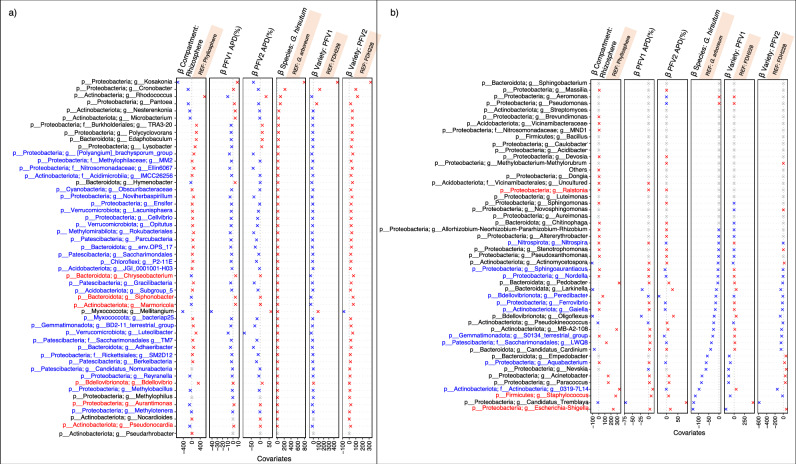
*β*-coefficients returned from the GLLVM procedure for covariates considered in this study. **a** Those coefficients that are positively associated with the microbial abundance of a particular species are represented in red color whilst those that are negatively associated are represented with blue color, respectively. Where the coefficients are non-significant, i.e., the 95% confidence interval crosses the 0 boundary, they are grayed out. Since the collation of OTUs was performed at Genus level, all those OTUs that cannot be categorized based on taxonomy are collated under “Others” category. For categorical variables, one level acts as a reference and is shown with an annotation of “REF” next to it. Note that we have considered running the algorithm on top 100 most abundant genera (including “Others”). **b** Continuation of results for $\beta -$ coefficients for various covariates returned from the GLLVM procedure, and for the remaining genera not shown in (**a**).

The Generalized Linear Latent Variable Model (GLLVM) procedure was utilized to regress microbial abundances against different sources of variability ((variety, compartment, and APD). This then gave β-coefficients for each covariate, whether they are positively or negatively associated with microbial abundance (Fig. 6). For the columns containing β-coefficients for PFV1 APD (%), and PFV2 APD (%), the genera indicated in blue color are associated with most disease suppression in both PFV1 and PFV2, respectively. Conversely, the ones indicated in red color are deemed not vital in disease suppression. Most of the genera associated with disease suppression belong to the rhizosphere rather than the phyllosphere. Among the phylum *Proteobacteria*, the noteworthy genera participating in disease suppression include genus *MM2, [Polyangium]_brachysporum_group, Ellin6067, Noviherbaspirillum, Ensifer, Cellvibrio, genus SM2D12, Reyranella, Methylobacillus, Methylotenera, Sphingoaurantiacus*, and *Aquabacterium*. The phylum *Actinobacteria* contains the genera *IMCC262566* as the one suppressing CLCuD. Other distinct genera from various phyla include *Peridibacter, Obscuribacteraceae, Lacunisphaera, Opitutus, Rokubacteriales, Parcubacteria, Saccharimondales, P2-11E (Chloroflexi), Gracilibacteria*, etc.

### Distinct phyllospheric and rhizospheric bacterial genera responsible for CLCuD suppression

**Fig. 7:**
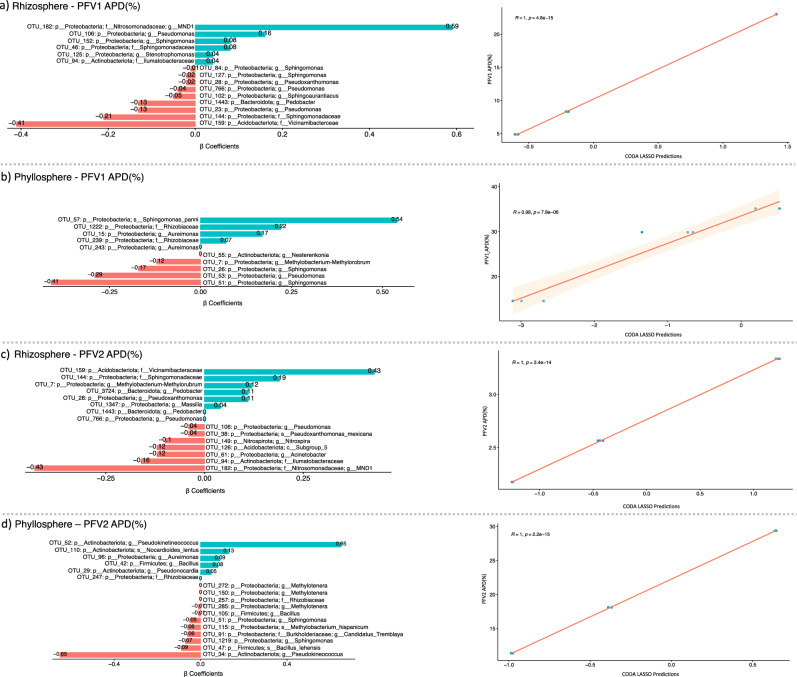
Non-zero *β*-coefficients returned from the CODA-LASSO procedure as two disjoint sets, those that are positively associated with APD (%) represented as green bars, and those that are negatively associated with APD (%) represented as red bars. The microbial fractions were applied on two varieties of *G. hirsutum*, PFV1, and PFV2, with the fractions extracted from both rhizosphere and phyllosphere with the combinations given as (**a**) rhizosphere PFV1, (**b**) phyllosphere PFV1, (**c**) rhizosphere PFV2, and (**d**) phyllosphere PFV2, respectively. The regression plots on the right side serve as a quality of fit plots with *R* values providing a means to assess whether the algorithm was able to find two sets or not.

Next, the CODA LASSO approach was used to identify the minimal subsets of bacterial genera that are associated with APD (%). Similar to the GLLVM procedure, β-coefficients are returned for microbial genera, however, by virtue of LASSO penalty in the CODA LASSO approach, some of the β-coefficients are returned as zero (those that do not have any association) resulting in two non-zero subsets: positively associated; and negatively associate. These are shown in Fig. 7. For PFV1 (partially tolerant variety), the bacterial genera suppressing the CLCuD and belonging to the rhizospheric core microbiome include *Vicinamibacterceae, Sphingomonadaceae, Pseudomonas, Pedobacter, Sphingoaurantiacus, Pseudoxanthomonas*, and *Sphingomonas (*Fig. 7a). For PFV2 (susceptible variety), however, the rhizospheric bacterial genera associated with disease suppression include MND1 from phylum *Proteobacteria, Ilumatobacteraceae, Acinetobacter, Acidobacteriota Subgroup 5, Nitrospira, Pseudomonas* and *Pseudoxanthomonas_mexicana* (Fig. 7c).

Similarly, the individual bacterial genera in the phyllospheric core microbiome associated with CLCuD suppression in PFV1 include *Sphingomonas, Pseudomonas, Methylobacterium-Methylorubrum*, and *Nesterenkonia* (Fig. 7b). In PFV2, the phyllospheric bacterial genera include *Pseudokineococcus, Bacillus lehensis, Sphingomonas, Candidatis tremblaya, Methylobacterium hispanicum, Bacillus, Methylotenera*, and *Rhizobiaceae* (Fig. 7d).

### RNA-seq: DEG and Gene Ontology analysis for interspecies and intraspecies microbiome transplants

**Fig. 8:**
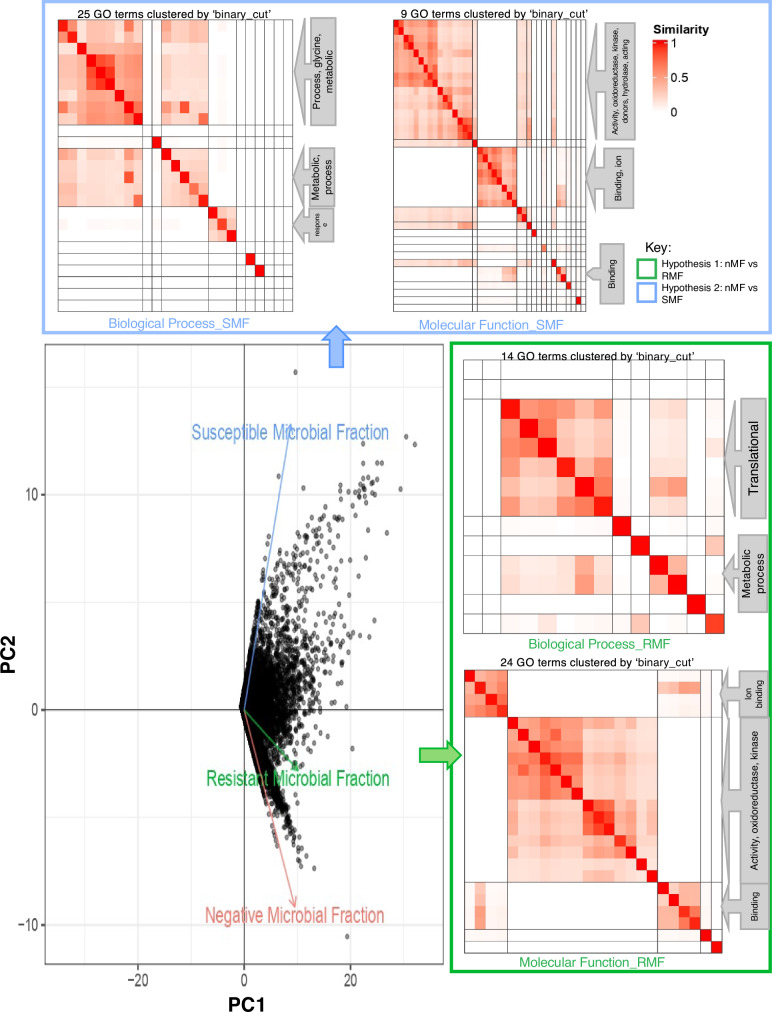
Transcriptomics profile of microbiome transplants. The lower left figure shows PCA plot of genes (shown as black points) with arrows pointing to the three different groups, as obtained from CummeRBund. The figures on the right and top are the clustered similarity plot of GO terms for each of the transplants represented as a heatmap. This is done separately for GO terms categorized under “Biological Processes” and “Molecular Functions”.

Figure 8 presents a comparative analysis of Gene Ontology (GO) terms for differential genes under two comparisons: *no microbial fraction* (nMF) vs *resistant microbial fraction* (RMF); and nMF vs *susceptible microbial fraction* (SMF). RMF group is the partially tolerant PFV1 *G. hirsutum* group transplanted with rhizospheric microbiome from completely tolerant *G. arboreum*. The comparison of the RMF group with the negative control *G. hirsutum* group is termed Hypothesis 1. For Hypothesis 1 (nMF vs RMF), which pertains to interspecies rhizospheric microbiota transplantation, Fig. 8 examines GO terms categorized under *Biological Processes* and *Molecular Functions*. For RMF, key biological processes affected include translational and metabolic processes, with 9 GO terms clustered using the “binary_cut” method. For RMF, key molecular functions include changes in oxidoreductase activity, kinase activity, and various binding activities, with 25 GO terms clustered. For Hypothesis 2 (nMF vs SMF), which covers the intraspecies rhizospheric microbiota transplantation, the figure similarly assesses GO terms associated with biological processes and molecular functions. For SMF, key GO terms associated with biological processes identify changes in glycine metabolic processes and general metabolic responses, with 14 GO terms clustered. For SMF key molecular functions show alterations in oxidoreductase activity, kinase activity involving donors, hydrolase activity, and ion binding, with 24 GO terms clustered. This analysis elucidates the specific GO terms that are significantly impacted in the different microbiome transplants, highlighting the functional and biological shifts occurring under each condition.

**Fig. 9:**
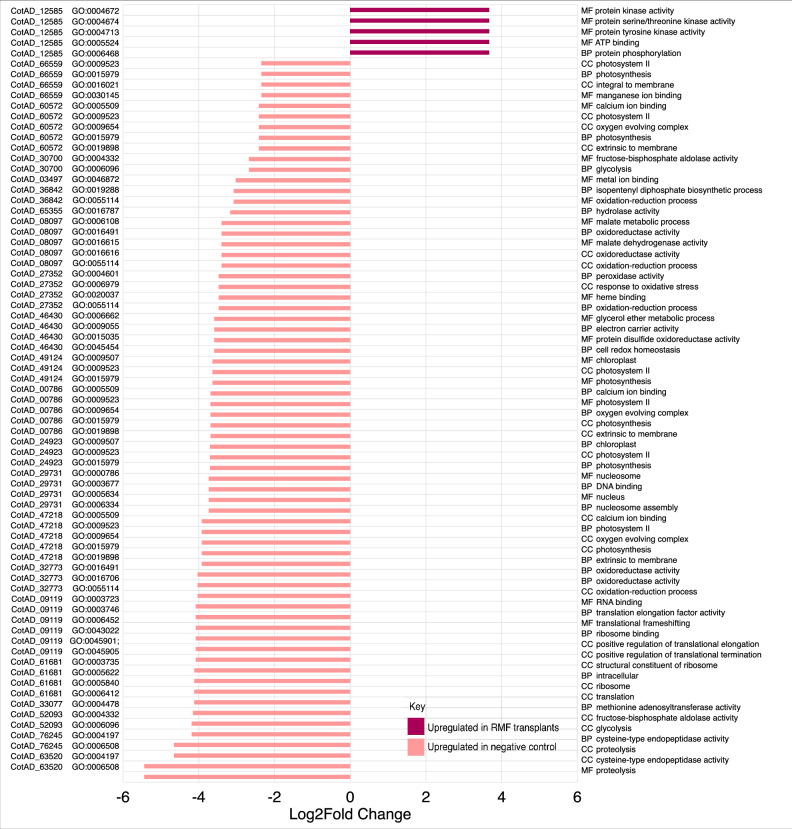
Log2fold change in gene expression for hypothesis 1, nMF vs RMF. **a** Log2fold change in specific genes upregulated in negative control and RMF transplants accompanied by original gene IDs, GO IDs, gene ontologies, and functional annotations. **b** Gene expression of specific genes upregulated in negative control and RMF transplants with significant differences shown.

The summary of RNA Seq results is given in Supplementary Data 1. For differentially expressed genes in each hypothesis, relevant GO IDs and original gene IDs from the BGI *Gossypium hirsutum* reference genome (www.cottongen.org) are also provided. Figure 9 depicts the significantly differentially expressed genes in the control group versus the interspecies rhizospheric microbiota transplant group in which *G. hirsutum* has been transplanted with *G. arboreum’s* rhizospheric microbiota. Key genes involved in stress response can be seen to be upregulated, including those encoding protein kinases, protein serine/threonine kinases, and protein tyrosine kinases. The molecular functions of ATP binding and protein phosphorylation have been observed to be significantly upregulated in interspecies transplants as well. The relative up and downregulated genes in intraspecies rhizospheric microbiota transplantation, nMF versus SMF, are given in Supplementary Figs. 10 and 11. The key upregulated biological processes in intraspecies microbiome transplants include phytochelatin biosynthetic process, photosynthesis light reaction, and tRNA processing.

The MDS plot for the samples is given in Supplementary Fig. 14. A deeper look into both hypothesis 1 and 2, i.e., nMF vs RMF and nMF vs SMF, respectively, shows predominant downregulation. The singular enrichment analysis profiles obtained from AgriGo.v2 for both hypotheses are given in Supplementary Figs. 15 and 16.

## Discussion

In this study, we have evolved the approach to CLCuD suppression from applying a few bacterial species to transplanting the entire microbiota. Whilst the previously published study^3^ was based on the application of candidate bacterial species to suppress CLCuD, it limited the full spectrum of interactions and benefits that naturally occurring microbial communities provide. Similarly, single-strain Plant Growth Promoting Rhizobacteria application suffers from unstable colonization and unprecedented competition involved with the residential taxa of the host microbiomes^14^. Therefore, whole microbiota transplantation leverages the entire microbial community, preserving the complex interactions among various bacterial species. This approach ensures a more robust enhancement of disease resistance, as it mimics the natural microbial environment more closely^15^.

From a practical standpoint, the main goal of any kind of suppression strategy utilizing microbiome modulation lies in developing biocontrol agents without hassle. The SA-producing bacteria in Aqueel et al.^3^ were isolated after a time-consuming step involving bacterial isolations from four different plant compartments (leaf epiphyte, leaf endophyte, rhizosphere, and root endophytes). This was followed by HPLC-based screening of individual isolates for phytohormone production. For the identified SA-producing bacterial strains, a minimal medium was designed prior to application for disease incidence assays. All these steps are avoided in the present approach where the microbiome is directly extracted using a single centrifugation-based strategy.

The comparative microbiome analysis of the rhizosphere and phyllosphere of *G. arboreum* and *G. hirsutum* revealed distinct bacterial genera uniquely associated with each species (the molecular insights into the basis of natural CLCuD resistance in the selected cotton species are given in the Supplementary Discussion). The rhizosphere of CLCuD-resistant *G. arboreum* (Rhi.RMF) appeared to harbor selective beneficial bacterial genera which, when transplanted onto susceptible host species *G. hirsutum*, imparted not only disease suppression but enhanced growth rate as well. The presence of *Rhizobium* in Rhi.RMF, a well-known nitrogen-fixer^16^, is such an example to which the higher growth rate of RMF transplants can be attributed. *Methylophilus*, another bacterial genus upregulated in *G. arboreum*, plays a significant role in plant growth promotion by utilizing reduced carbon compounds^17^, along with *Rhizobiales*, the plant partners known for providing essential nutrients, phytohormones, and precursors for essential plant metabolites^18^. Simultaneously, the suppression of CLCuD in Rhi.SMF *G. hirsutum* PFV2 plants, transplanted with their own microbiome, authenticates the views of Berendsen et al.^19^ again, that the plant calls beneficial microorganisms to its aid when its aerial parts are under attack by pathogens. As a result, it becomes evident that the susceptible *G. hirsutum* PFV2 plants harbor certain beneficial bacterial genera that have the potential to induce disease tolerance. This effect is especially significant when the bacterial populations are intentionally increased or concentrated within the plant compartments transplanted with their own microbiomes. In such cases, the higher density of these beneficial microbes appears to enhance their ability to promote disease tolerance in the host plants.

The selection of similar bacterial genera has been observed in the core microbiome of the host *G. arboreum* plant. Since the plant’s core microbiome is known for harboring crucial bacterial candidate species responsible for carrying out functions of high value for the host^20^, the core microbiome of *G. arboreum* also appears to have selected certain crucial bacterial species. The selected species are known to contribute significantly to disease suppression under severe biotic stress. Planctomycetota is one dominant phylum within the microbial community of disease-suppressive soils^21^. The presence of MBNT15 is also indicative of the disease suppressive properties of the *G. arboreum* rhizosphere. It is a distant relative of Desulfobacterota and is known for its role in dissimilating iron reduction and utilizing nitrogen and sulfur compounds through both aerobic and anaerobic respiration^22^. A selection pressure of *G. arboreum* core microbiome towards *Acidobacteriota* and *MBNT15* and the resulting reduction in CLCuD is also supported by the action of these phyla in brown rice, where they have been reported to play a role in enhanced concentrations of mineral elements while reducing the accumulation of hazardous heavy metal cadmium^23^.

*Vicinamibacterceae*, found to be positively associated with disease suppression, is a rare and recently known genus of Acidobacteria, associated with presence in rhizospheric soil of the plant groups demonstrating higher growth and yield^24^. *Sphingomonas* has been broadly associated with disease suppression in plants and has been reported in several studies including Innerebner et al.^25^, where it has been found to diminish the pathogen growth in *Arabidopsis thaliana. Pseudokineococcus* is another rare genus of *Kineococcus*, but unlike *Kineococcus*, the reports of *Pseudokineococcus* remain too scarce. *Kineococcus rhizosphaerae sp*. has been reported to be found in plant rhizospheric soil^26^, but *Pseudokineococcus basanitobsidens* has only been reported to be found in volcanic rock^27^. The identification of the presence of these genera in the core microbiome of *G. arboreum* species opens new gates for detailed research in the functions of these bacterial genera in relation to plant health against viral pathogens and can be a strong way forward towards sustainability.

The application of exogenous SA was also found to effectively reduce the incidence of CLCuD in treated groups. This can be attributed to the activation of the plant’s immune response, specifically systemic acquired resistance. When infected by biotrophic pathogens, plants activate internal defense networks in response to the SA they produce as part of their natural defense mechanism. SA acts through NPR1, a key player in signaling plant defense against pathogens^28^. NPR3 and NPR4 interact with SA by mediating NPR1 degradation or directly binding to SA to modulate NPR1 interaction^29^. After infection, SA levels increase in the plant, enhancing NPR1 degradation, allowing its monomeric form to migrate to the nucleus and interact with TGA proteins to boost SA-mediated PR gene expression^30^. Foliar SA also alters phyllospheric microbial diversity, enriching it with microorganisms possessing antibiotic and xenobiotic degradative properties, further inducing resistance^31^.

For the experiment of phyllospheric microbiota transplantation, the overall disease suppression did not surpass the disease suppression response as exhibited by rhizospheric MF transplants. A possible improvement in the experiment could be the application route of phyllospheric microbiota, which should have been applied both through soil drench and foliar spray method, to ensure proper colonization of all plant compartments. In order to obtain a proper insight into the actual functional pathways in the host plant that are affected by the transplantation of the microbiome, this study utilizes proper functional profiling using both the *16S rRNA* data and the mRNA transcriptomic data for deeper analysis.

Functional profiling inferred from *16S rRNA* gene sequencing data is often unreliable due to the incompleteness of the database of the reference genomes for which functional profiles are readily available. However, of all the commonly used prediction tools, PICRUSt2 still remains the best choice by virtue of its sensitivity to detect ubiquitous function^32^. Furthermore, the recent release of PICRUSt2 results in 10-fold increase in database size(~20,000 genomes in PICRUSt2 as opposed to ~2000 in PICRUSt1) which results in marked improvement of the prediction quality. Indeed, in our dataset, only 99 out of 4139 ASVs were above the max NSTI cut-off of 2.0 (internal criteria for returning a match against a reference genome) and were not assigned to the reference database. With 97.6% of the total ASVs matching in this study, there is an increased confidence in the utility of the functional data for downstream statistical analyses.

The enhanced accuracy in functional profiling, therefore, allows for a more robust analysis of the microbial community’s role in plant microbiome compartments, as indicated by differential pathway upregulation. Functionally, the upregulation of the pathways for peptidoglycan biosynthesis, starch degradation, and hydroxyacetophenone degradation in *G. arboreum* rhizosphere (Rhi.RMF), indicates the plant growth-promoting role of the bacterial community involved. The MFs from the partially tolerant variety PFV1 (Rhi.pRMF) and susceptible PFV2 (Rhi.SMF) of *G. hirsutum*, on the other hand, have a highly upregulated pathway of Tryptophan biosynthesis. Since tryptophan is a precursor of Indole Acetic Acid, the main auxin in plants, the upregulation of this pathway indicates that the rhizospheric microbiome helps *G. hirsutum* in combating high biotic stress.

The pathway analysis of the Phyllospheric MFs indicates the role of phyllospheric bacteria in plant growth promotion through the upregulation of the starch biosynthesis pathway in FDH228 phyllosphere (Phy.RMF). The distinct role of the phyllospheric bacteria is also explained by the significant functions being performed by the candidate bacteria in the phyllosphere of the partially tolerant *G. hirsutum* variety, PFV1 (Phy.pRMF). The phyllosphere is seen to perform the function of Vitamin B6 and taurine degradation as sources of carbon and nitrogen for growth^33^. Moreover, the upregulation of phospholipase (PLA) synthesis indicates the coping mechanism in *G. hirsutum* in the presence of biotic stress and pathogen attack, given that PLAs play a role in plant signal transduction, including their response to factors like auxin-induced growth, pathogens, and elicitors^34^. The superpathway of N-acetylglucosamine, N-acetylmannosamine, and N-acetylneuraminate has also been upregulated in the phyllosphere of *G. hirsutum* (PFV1 and PFV2). Amino sugars are integral elements of the cell surface structures of *Escherichia coli* and can serve as both carbon and nitrogen sources. N-acetylglucosamine, mannosamine, and neuraminic acid are all transportable into the cell and subject to metabolic processes. These three dissimilation pathways ultimately intersect at N-acetylglucosamine-6-phosphate^35^.

Both interspecies and intraspecies applications of single-strain bacteria have been employed by researchers in the past for plant growth promotion, plant breeding, and disease suppression. In order to have a clear insight into the changes occurring in the plant’s gene expression patterns when transplanted with another plant’s microbiome, a valuable tool is a transcriptomic analysis. The current study, therefore, details the transcriptomic analysis of the complete rhizospheric transplantations for CLCuD suppression, within the *G. hirsutum* varieties (intraspecies), and among *G. hirsutum* and *G. arboreum (*interspecies) as well.

Cell signaling pathways are essential for regulating plant growth, development, and responses to environmental changes. Our results reveal protein phosphorylation as the key biological process upregulated in RMF (interspecies) transplants. Protein phosphorylation, a reversible post-translational modification, plays a critical role in ensuring the specificity and robustness of these signals. It is the most common post-translational modification, occurring on serine, threonine, and tyrosine residues, and is facilitated by protein kinases while being reversed by protein phosphatases^36^. This modification can alter a protein’s conformation, activity, interactions, stability, and localization. Recent research highlights the importance of protein phosphorylation in various aspects of plant life, such as growth, development, stress responses, and most importantly, phytohormone homeostasis and recruitment^37^. The upregulation of protein tyrosine-kinase, protein serine/threonine kinase, and protein kinase indicate a higher level of signal transduction taking place within the RMF transplants, explaining the possible molecular basis of the enhanced disease suppression and growth in the transplants.

Another molecular function upregulated in RMF transplants includes ATP-binding. ATP-binding in plants is mediated by the ATP-binding cassette (ABC) protein superfamily. ABC protein is responsible for the transmembrane transportation of critical compounds like polar auxins in order to impart the plant with xenobiotic detoxification, stomatal function, and disease resistance^38^. The upregulation of ATP-binding is also explanatory for the resulting disease suppression of the Rhi.RMF transplants group. The transcriptomic profiling of the interspecies rhizospheric transplants strongly indicate the mediation of signal transduction in host plants for suppression and response to disease attack.

Through the in-depth analysis of the natural microbial community and their functional pathways involved in disease suppression and signal transduction in host plants, the presented study grounds the significance of microbiota transplantation in disease suppression and plant growth. The technique can prove to be a sustainable alternative to traditional practices for pest management and crop productivity.

## Methods

### Experimental design

Two varieties of *Gossypium hirsutum* and one variety of *Gossypium arboreum* were chosen for carrying out all the research work based on their relative resistance to the CLCuV. The varieties of *Gossypium hirsutum* included PFV1 (partially tolerant to CLCuV) and PFV2 (completely susceptible to CLCuV). The variety chosen for *Gossypium arboreum* was FDH228 *desi cotton* (resistant to CLCuV). The seeds for PFV1 and PFV2 were obtained from Four Brothers Research Farm, Lahore, and the seeds for FDH228 were obtained from Ayub Agriculture Research Institute, Faisalabad. All the seeds were linted when received and were delinted freshly with concentrated sulfuric acid before sowing.

In March 2021, single batch of all three varieties, containing 30 plants each, was sown in Net House 1 (Supplementary Fig. 17a) of Botanical Garden at Forman Christian College (A Chartered University), Ferozepur Road, Lahore (31.5204°N, 74.3587°E). Viruliferous *Bemisia tabaci*, locally referred to as the whitefly, were previously reared in the net house so the net house served as a whitefly hotspot. These plants were grown till the flowering stage and were used for rhizospheric and phyllospheric microbial fraction (MF) extraction.

The microbial fractions extracted were then applied to test plants. The test groups were classified according to the MF type. The MF extracted from the CLCuV-completely resistant *desi cotton* FDH228 was termed as RMF (Resistant Microbial Fraction). The MF extracted from the CLCuV partially tolerant variety PFV1 was termed as pRMF (partially-Resistant Microbial Fraction). The MF extracted from the CLCuV completely susceptible variety PFV2 was termed as SMF (Susceptible Microbial Fraction). The batch to which no MF was applied served as a negative control and was termed as nMF (no Microbial Fraction) and the batch to which 0.4 mg/mL SA was applied was termed as SA (salicylic acid). Five batches of 30 plants each for each of the three varieties were prepared for the application of RMF, pRMF, SMF, SA, and nMF, respectively. Test group abbreviations and MF application details are given in Supplementary Table 1 and Supplementary Fig. 1, respectively.

### Microbial fraction preparation

For the preparation of rhizospheric MF, the soil of the plants of each variety grown in Net House 1 was sampled. The plant was uprooted and the bulk soil was removed with a trowel. The roots were shaken to remove the loose soil, leaving behind only the tightly adhering soil to the root, the rhizospheric soil. The root and soil were transferred to clean, dry, and labeled polythene (autoclave) bags and carried to the laboratory on ice. For Phyllospheric MF, the leaves from each plant were picked and transferred to clean, dry, and labeled polythene (autoclave) bags and carried to the laboratory on ice. The roots along with rhizospheric soil were then transferred to autoclaved and dried 1 L Erlenmeyer flask containing 500 mL of 1X TE buffer. The soil and root suspension were mixed in a shaking incubator for 30 min at 30 °C at 150 rpm, after which the suspension was filtered through 2.5 µm sterile Whatman filter paper to remove the roots and debris. The filtered suspension was transferred to 1 L clean and dry centrifuge bottles and centrifuged for 5 min at 2000 rpm at 4 °C. The sedimented soil pallet was discarded and the supernatant was transferred to clean and dry 1 L centrifuge bottles and centrifuged for 30 min at 8000 rpm at 4 °C (Supplementary Fig. 18). The supernatant was discarded and the cell pallet was resuspended in 1X TE buffer (pH 8.0) for final application to the plants.

For the preparation of Phyllospheric MF, the leaves were removed from the stem and rinsed with distilled water gently to remove soil particles. In autoclaved and dried mortar, the leaves were finely crushed with pestle in the presence of 1X TE buffer. The leaf suspension was resuspended in 500 mL 1X TE buffer and shaken at 150 rpm for 30 min at 30 °C. The suspension was filtered through 0.5 µm sterile Whatman filter paper to remove leaf debris. The filtered suspension was transferred to 1 L clean and dry centrifuge bottles and centrifuged for 5 min at 2000 rpm at 4 °C. The sedimented leaf debris pallet was discarded and the supernatant was transferred to clean and dry 1 L centrifuge bottles and centrifuged for 30 min at 8000 rpm at 4 °C. The supernatant was discarded and the cell pallet was resuspended in 1X TE buffer for final application to the plants (Supplementary Fig. 19).

The bacterial community in each MF sample was then quantified using the CFUmL^−1^ method (Supplementary Data 2). The cell pallets collected for each MF were resuspended in 1 mL of sterile 1X TE buffer and serially diluted (three times for Rhi.MF samples and two times for Phy.MF samples). 0.1 mL of each MF was spread on half-strength tryptic soy agar plates and incubated for 24 h. The CFUmL^−1^ for each sample was calculated using the following equation from Tortora 2018^39^:

$${CFU}/{mL}=\frac{{Number\; of\; colonies}\times {Total\; Dilution\; Factor}}{{Volume\; of\; inoculant\; plated}}$$

### Seed delinting and sowing

Cotton seeds of both species, *Gossypium hirsutum* (PFV1 and PFV2) and *Gossypium arboreum* (FDH228), were delinted with 80% H_2_SO_4_ for ~1 min, followed by rinsing with 1 N NaOH for 1 min. The seeds were then given 3–4 washes with sterile distilled water and dried on a paper towel prior to sowing. The nursery soil was autoclaved twice at 121 °C for 40 min, with an interval of cooling down to room temperature. The sterilized and cooled soil was filled in small 6 in. pots (surface sterilized with ethanol), with 50 g soil in each pot and 4 seeds sown per pot. After germination, the seeds were allowed to grow into 7-day-old seedlings in a temperature-controlled room (Supplementary Fig. 17b) at 28 °C, with an artificially controlled photoperiod regime set at 16 h of light and 8 h of dark throughout the experiment.

### Microbial fraction and salicylic acid application

The rhizospheric and phyllospheric MFs were freshly prepared and were applied to 7-day-old seedlings by soil-drench method, 1 mL per gram of soil. To each 50 g pot, 50 mL of the MF was applied to all test groups on the same hour of the day. To the nMF test groups, sterile distilled water was applied simultaneously, whereas, to the SA test groups, 400 mgmL^−1^ sterile SA solution was applied in the form of a foliar spray. Both surfaces of the leaves were covered with spray. The plants were grown in climate-controlled conditions for a further 3 weeks.

### Cotton leaf curl virus inoculation

*Begomovirus-*infected whitefly, *Bemisia tabaci*, was reared in the net house on previously grown CLCuV susceptible *Gossypium hirsutum* plants. These plants were tested for *begomovirus* presence by amplification of β-satellite regions of *begomovirus* (Supplementary Tables 2 and 3) from plant leaf genomic DNA. The 4-week-old plants were shifted to the whitefly hotspot net house for viral inoculation (Supplementary Fig. 17c), keeping the completely randomized pot design intact. CLCuV infection was identified by amplification of β-satellite region (Supplementary Fig. 20).

### Cotton leaf curl disease incidence assays

All test plants were observed daily for 60 days post-viral inoculation for the appearance of CLCuD symptoms, and the number of diseased leaves and total leaves were recorded for each day. Percentage disease for each plant was calculated for each day using the equation:

$${\rm{Percentage}}\; {\rm{disease}}\left( \% \right)=\frac{{\rm{number}}\; {\rm{of}}\; {\rm{diseased}}\; {\rm{leaves}}}{{\rm{total}}\; {\rm{number}}\; {\rm{of}}\; {\rm{leaves}}}\times 100$$

After 60 days of the assay period, APD of all the replicates in each test group was calculated for each day using the equation:

$${\rm{Average}}\; {\rm{Percentage}}\; {\rm{Disease}}\; {\rm{on}}\; {\rm{each}}\; {\rm{day}}\left( \% \right)\\ =\frac{{\rm{Sum}}\; {\rm{of}}\; {\rm{Percentage}}\; {\rm{Disease}}\; {\rm{in}}\; {\rm{each}}\; {\rm{replicate}}}{{\rm{total}}\; {\rm{number}}\; {\rm{of}}\; {\rm{replicates}}}$$

For the Disease Severity Index, each plant was scored for CLCuD following the disease rating scale given by Akhtar et al. (2015)^40^. The number of diseased leaves and total leaves for each plant were counted each day for a span of 60 days post-CLCuV inoculation. For the calculation of Disease Severity Index, the following equation was used:

$${\rm{Disease}}\; {\rm{Severity}}\; {\rm{Index}}=\frac{{\rm{Number}}\; {\rm{of}}\; {\rm{Diseased}}\; {\rm{Leaves}}}{{\rm{Total}}\; {\rm{number}}\; {\rm{of}}\; {\rm{Leaves}}}\times 6$$

Where “6” is the maximum severity score according to Akhtar et al.^40^.

The Average Percentage Disease Assay and Disease Severity Index Assay are given in Supplementary Data 3.

### Statistical analysis of disease incidence assays

All the data was analyzed using Microsoft Excel (Microsoft Office 365) including chart development. All the statistical analyses were performed on IBM^®^ SPSS^®^ Statistics Version 25. The significant difference between test groups was calculated using one-way univariate analyses of variance (One-Way ANOVA test) and Tukey’s honestly significant difference (HSD) *post-hoc* test in IBM^®^ SPSS^®^ Statistics. The differences among test groups were considered significant at *p-value* less than 0.05 with significance values as: *p < 0.05, **p < 0.01, or ***p < 0.001.

### Bacterial diversity analysis of microbial fractions

#### Total DNA isolation and *16S rRNA* gene amplification

Total DNA was isolated from prepared microbial fraction (cell pallet) samples using FastDNA Spin Kit for Soil (MP Biomedicals) according to the manufacturer’s protocol. The hypervariable V3-V4 region of the *16S rRNA* gene was amplified using PCR reaction containing 12.5 µL DreamTaq GreenPCR Master Mix (Thermo Scientific), 1 µL each of 10 µM forward 341 F (**TCGTCGGCAGCGTCAGATGTGTATAAGAGACAG**CCTACGGGNGGCWGCAG) and reverse primers 805 R (**GTCTCGTGGGCTCGGAGATGTGTATAAGAGACAG**GACTACHVGGGTATCTAATCC), 1 µL each of mPNA and pPNA blockers, 2 µL total DNA (10 ng/ µL) and 6.5 µL of nuclease-free water. The PCR program was configured with 5 min of initial denaturation at 95 °C, followed by 35 cycles consisting of 1 min of denaturation at 94°C, 1 min of annealing at 55 °C and 30 s of extension at 72 °C. The final extension step was set at 72 °C for 10 min. The amplified PCR product was cleaned using PureLink PCR Purification Kit (Thermo Scientific). The cleaned PCR products were sequenced using paired-end metagenome amplicon sequencing on the Illumina® MiSeq Platform by Macrogen, South Korea.

### Bioinformatics and statistical analysis

We have used the traditional VSEARCH workflow to generate Operational Taxonomic Units (OTUs) on *n* = 18 samples, as done previously with the following modifications: (a) we classified the taxonomy of the OTUs using the recent SILVA SSU Ref NR database release v.138^41^; (b) we generated the rooted phylogenetic tree with the QIIME2 framework^42^; (c) we used PICRUSt2^43^ within the QIIME environment to recover KEGG enzymes and MetaCyc pathway predictions; and (d) OTUs were generated by using 99% similarity threshold, and as a pre-processing step, the reads were quality trimmed at Phred quality score of 20 and kept with a minimum length of 200 bp with a total of 1,342,501 total reads for *n* = 18 samples, with 1,335,148 reads making into the final analysis after error correction using BayesHmmer, and overlapping paired-end reads with Pandaseq, a strategy that reduces noise in the reads significantly^44^. As a sanity check, we also generated Amplicon Sequencing Variants (ASVs) using DADA2 in QIIME2, however, the summary statistics resulted in low ASVs numbers and this analysis was not pursued further. The final dimensions of the tables are then as follows: *n* = 18 × *P* = 4139 (OTUs) table; *n* = 18 × *P* = 10,543 (KEGG KOs) table; *n* = 18 × *P* = 483 (MetaCyc Pathways) table. The samples-wise summary statistics for OTU table is [Min: 12,767, 1st Quartile: 18,689; Median: 26,269; Mean: 35,134; 3rd Quartile: 55,774; and Max: 73,776.

QIIME2 was also used to generate a final BIOM file that combined abundance information with the new taxonomy, which along with the new phylogenetic tree, and the metadata was used for the downstream statistical analysis in R. As a pre-processing step in R, we removed typical contaminants such as *Mitochondria*, and *Chloroplasts*, as well as any OTUs that were unassigned at all levels, as per recommendations given at https://docs.qiime2.org/2022.8/tutorials/filtering/. The R’s vegan package^45^ was used for diversity analyses. For alpha diversity measures we have used (after rarefying to minimum library size): (i) *Shannon entropy*—a commonly used index to measure balance within a community; (ii) *Chao1 richness*—the estimated number of species/features in a rarefied sample. We have used R’s aov() function to calculate the pair-wise analysis of variance (ANOVA) *p*-values which were then drawn on top of alpha diversity figures.

Vegan package was also used to perform PERMANOVA analyses to see if the microbial or functional community structures can be explained by different sources of variability. To find genera (OTUs collated at genus level), KEGG KOs, and MetaCyc pathways that are significantly different between multiple conditions, we used the DESeq2 package^46^ with the adjusted *p*-value significance cut-off of 0.05 and log2 fold change cut-off of 2. This function uses negative binomial GLM to obtain maximum likelihood estimates for log fold change of abundance between the two conditions. Then Bayesian shrinkage is applied to obtain shrunken log fold changes subsequently employing the Wald test for obtaining significances. The final expression levels of resulting discriminating features were then drawn after using TSS + CLR (total sum scaling followed by centralized log ratio transform) normalization.

### Core microbiome analysis

The approach first ranks the OTUs by occupancy and then calculates the minimal occupancy threshold dynamically by learning from the data. The ranking of OTUs is done using a combination of two metrics: *site-specific occupancy* (whether samples are grouped by different treatment groups, namely, *Phyllosphere* and *Rhizosphere*); and replicate consistency (whether the OTUs are consistent across replicates in the above treatment group). After ranking the OTUs, the subset of core taxa is constructed incrementally by adding one OTU at a time to the core set of OTUs, from highly prevalent to lowly prevalent ones. The contribution of the core subset to beta diversity is then calculated every time a new OTU becomes member of the core set using the Bray-Curtis distance in the equation, $C=1-\frac{B{C}_{{core}}}{B{C}_{{all}}}$. The original authors have specified a threshold at which the core subset construction stops, i.e., where the addition of an OTU does not cause more than 2% increase in the explanatory value by Bray-Curtis distance. Independently, a neutral model^47^ is fitted to the “S” shaped abundance-occupancy distributions inform the OTUs that are likely selected by the environment. These are obtained as those that fall outside the 95% confidence interval of the fitted model, and are inferred to be deterministically assembled, rather than neutrally selected, with those that are above the model selected by the host environment (represented by red color), and those points below the model are dispersal limited (represented by blue color). The taxonomy tree of the core microbiome across different breeds and treatment groups were drawn using the R’s metacoder package^48^.

To find the relationship between individual microbes and sources of variability, we have used GLLVM^49^. GLVMM extends the basic generalized linear model that regresses the mean abundances ${\mu }_{{ij}}$ (for $i$-th sample and $j$-th microbe) of individual microbes against the covariates ${x}_{i}$ by incorporating latent variables ${u}_{i}$ as $g({\mu }_{{ij}})={\eta }_{{ij}}={\alpha }_{i}+{\beta }_{0j}+{{\boldsymbol{x}}}_{i}^{T}{{\boldsymbol{\beta }}}_{j}+{{\boldsymbol{u}}}_{i}^{T}{{\boldsymbol{\theta }}}_{j}$, where ${{\boldsymbol{\beta }}}_{j}$ are the microbe specific coefficients associated with individual covariate (a 95% confidence interval of these whether positive or negative, and not crossing 0 boundary gives directionality with the interpretation that an increase or decrease in that particular covariate causes an increase or decrease in the abundance of the microbe), and ${{\boldsymbol{\theta }}}_{j}$ are the corresponding coefficients associated with latent variable. ${\beta }_{0j}$ are microbe-specific intercepts, whilst ${\alpha }_{i}$ are optional sample effects which can either be chosen as fixed effects or random effects.

REF refers to a reference that gets dropped in the regression model when coding for categorical parameters. To model the distribution of individual microbes, we have used Negative Binomial distribution. Additionally, the approximation to the log-likelihood is done through variational approximation (VA) with final sets of parameters in glvmm() function being family = “negative.binomial”, method = “VA”, and control.start = list(n.init = 7, jitter.var = 0.1) seemed to fit well.

To find a minimal subset of OTUs that changed against APD (%) in PFV1, and PFV2, we used the CODA LASSO of the form ${y}_{i}={\beta }_{0}+{\beta }_{1}{\mathrm{log}}\left({x}_{1i}\right)+\ldots +{\beta }_{j}{\mathrm{log}}({x}_{{ji}})+{\epsilon }_{i}$ (for $i$-th sample and $j$-th feature, with ${x}_{{ji}}$ being the abundance of OTU, and where the outcome ${y}_{i}$ is a continuous variable (APD %). The model uses two constraints: (a) $\sum _{k\ge 1}{\beta }_{k}=0$ (i.e., all $\beta$-coefficients sum up to zero) which makes the algorithm invariant by always returning two disjoint sets of features, i.e., those that have positive association, and those that have negative association, respectively; and (b) the optimization function incorporates a LASSO shrinkage term $\lambda {\sum }_{k\ge 1}|{\beta }_{k}|$ as ${\sum }_{i=1}^{n}({y}_{i}-{\beta }_{0}-{\beta }_{1}{\mathrm{log}}({x}_{1i})-\ldots -{\beta }_{j}{\mathrm{log}}{({x}_{{ji}})})^{2}+\lambda {\sum }_{k\ge 1}|{\beta }_{k}|$ subject to ${\sum }_{k\ge 1}{\beta }_{k}=0$. Here, $\lambda$ is the penalization parameter, and forces some of the ${\boldsymbol{\beta }}$-coefficients to go zero, particularly those that do not have a relationship with the OTUs and serve as a means to do variable selection. We have used coda_glmnet() function from R’s coda4microbiome package^50^. We have used the top 100 most abundant OTUs in the CODA-LASSO model and have run the algorithm separately for rhizospheric and phyllospheric communities.

### Transcriptome analysis of cotton microbiome transplants

A separate pot analysis was set for transcriptome analysis of the cotton microbiome transplants. Three groups were chosen based on the results obtained from disease suppression pot analysis, namely PFV1.RMF (PFV1 *G. hirsutum* plants transplanted with rhizospheric MF from *G. arboreum* FDH228), PFV1.SMF (PFV1 *G. hirsutum* plants transplanted with rhizospheric MF from PFV2 *G. hirsutum*), and PFV1.nMF (the negative control). Each group consisted of 30 plants and was grown in the same way as described in sections 4.2–4.6. For RNA isolation, leaf samples were picked from 10 plants in each group, snap-frozen in liquid nitrogen, and pooled, giving three representative replicates from each transplant group. The leaves were wiped clean with 1% SDS solution and absolute ethanol, followed by rinsing with sterile distilled water prior to sampling to reduce the presence of any contaminants in the sample. Sampling was carried out at the same hour for all groups on the same day, 25 days post viral inoculation, and were carried to the laboratory in sterile vials frozen in liquid nitrogen. Total RNA isolation was carried out using Invitrogen Plant RNA Purification Reagent Cat. No.: 12322-012, according to the manufacturer’s protocol.

The paired-end transcriptome resequencing was carried out by Macrogen Inc. (South Korea), using Illumina® NovaSeq platform and TruSeq stranded mRNA library prep kit (raw data statistics are given in Supplementary Table 4). The raw reads were quality trimmed through sickle (https://github.com/najoshi/sickle) using Phred quality cutoff of 20 and retained reads with the minimum read length of 50 bp. The reference genome of *Gossypium hirsutum* (AD1) “TM-1“ genome was downloaded from CottonGen Cotton Resource Database (https://www.cottongen.org/species/Gossypium_hirsutum/bgi-AD1_genome_v1.0) along with the detailed annotation data for all chromosomes in the GFF3 format. The annotation contained information about genes, transcripts, exons, and other genomic features. We then followed the recommendations according to the RNA-seq protocol by Trapnell et al.^51^. Differential gene and transcript expression analysis of RNA-seq experiments with TopHat and Cufflinks. Briefly, the quality-trimmed reads were aligned against the reference genome using the splice-aware aligner, TopHat v2.1.1, and the above annotation file was included during the alignment process. The summary read statistics on sample-by-sample basis are given in Supplementary Table 5.

The aligned reads in BAM format from each sample were then used as input to Cufflinks v2.2.1 to perform transcriptome assembly. Within the Cufflinks framework, the assembled transcripts were merged together using cuffmerge, whilst coding for information related to experimental conditions including biological replicates. This was followed by using cuffdiff to perform differential analyses. Here, Fragments per Kilobase of exon model per Million reads mapped (FPKM) were estimated for each gene. The output generated by cuffdiff was then further analyzed using CummeRbund^52^. Additionally, custom-made R scripts were developed to enhance data representation in downstream processes. Differentially expressed genes were identified by calculating the fragments per kilobase of transcript per million (FPKM) value and read count of each gene. These were determined based on a log2 fold change of at least 1.5 and a false discovery rate of *p* < 0.05.

Next GO annotations (BGI_Gossypium_hirsutum_gene.GO.result.gz) were downloaded from https://www.cottongen.org/species/Gossypium_hirsutum/bgi-AD1_genome_v1.0 to obtain GO IDs for differentially expressed transcripts. These GO IDs were then subjected to enrichment analysis using R’s simplifyEnrichment package^53^. Briefly, the package enables the clustering of redundant GO functional terms into separate groups using the similarity between GO IDs through a “binary cut” method. The resulting terms show more consistent similarities within clusters, and more mutual exclusion between clusters.

### Reporting summary

Further information on research design is available in the Nature Portfolio Reporting Summary linked to this article.

## Supplementary information

Supplementary Information

Description of Additional Supplementary Files

Supplementary Data 1

Supplementary Data 2

Supplementary Data 3

Supplementary Data 4

Reporting summary

## Data Availability

Sequence data for amplicons is available from the sequence read archive (SRA) database under Bioproject No. PRJEB68138 (Supplementary Data 4.xlsx) whilst sequence data for transcriptomics is available from SRA database under Bioproject No. PRJEB78371 with details of the samples provided in Supplementary Data 1.xlsx. The disease severity data (Fig. 3) is available in Supplementary Data 3.xlsx. Supplementary Figs. 21–23 are associated with Supplementary Results. The uncropped and unedited gel image for Supplementary Fig. 20 is provided in Supplementary Fig. 24. The data for all other figures is provided in the associated Zenodo (10.5281/zenodo.14912808) repository.

## Code availability

Some of the code used to generate the results are part of microbiomeSeq (https://github.com/umerijaz/microbiomeSeq), whilst other codes are available at http://userweb.eng.gla.ac.uk/umer.ijaz#bioinformatics. Furthermore, the data tables and code (adapted from above, and specific to this study) used in the analysis of amplicons and transcriptomics data are available in the associated Zenodo (10.5281/zenodo.14912808) repository.
