# Supplementary material for: Microbiota transplantation for cotton leaf curl disease suppression—core microbiome and transcriptome dynamics
Source: Commun Biol. 2025 Mar 6;8:380. doi: 10.1038/s42003-025-07812-7 (PMC11885576; doi:10.1038/s42003-025-07812-7)
Supplement: Supplementary file 1 — Supplementary Information [file 42003_2025_7812_MOESM1_ESM.pdf]

Supplementary Information

Supplementary Note 1

The first epidemic of the inimical CLCuD in Pakistan was recorded in 1990, which resulted in a total economic loss of approximately USD 5 billion to the country until 1997. The causative agent of CLCuD, the *begomovirus,* has been characterized into five different species and causes severe stunting of growth, vein thickening and curling of leaves, limiting photosynthetic surface of the leaf. This reduces cotton yield by around 15 to 17% [1]. The controlling strategies for CLCuD have been revolving around either crop management practices (like the use of insecticides to limit white fly infestation) or development of resistant cotton cultivars, both of which do not provide permanent solutions to the problem, as the virus is prone to rapid mutation. Several breeding studies were performed to develop CLCuD-tolerant cultivars [2-4], but none of the cultivars remained tolerant for more than 5 years, after which the *begomovirus* mutated into the Burewala strain.

A ray of hope for breeding scientists and agriculturists is the diploid cotton, *G. arboreum* and *G. herbaceum*, which are naturally tolerant to CLCuV [5]. *G. arboreum,* the cotton species native to Pakistan, is locally referred to as *desi cotton*. Despite being naturally immune to various biotic stresses, including CLCuV, the species yield cotton with very short fiber length, rendering it unsuitable for manufacture of good quality fabric. The other most grown species, *G. hirsutum*, yields cotton with a long fiber length, a quality much desired by the cotton industry, but is highly susceptible to biotic stresses including CLCuD.

Supplementary Results

Supplementary results 1: Culture-based quantification of bacterial community in Microbial Fraction samples in CFU/mL

The culture plates in nutrient medium for MF samples is shown in Supplementary Figure 21. The bacterial quantity (Supplementary Data 2) in each MF sample was quantified and Log_{10} $CFUmL^{-1}$ plotted for each (Supplementary Figure 22). The highest number of bacterial cells were recorded in Rhi.RMF with Log_{10} $CFUmL^{-1}$ at 5.94 which was significantly higher than all the phyllospheric MFs, Phy.RMF, Phy.pRMF and Phy.SMF.

The Log_{10} $CFUmL^{-1}$ for Rhi.SMF was also recorded to be significantly higher than its phyllospheric counterpart, Phy.SMF.

Supplementary results 2: Elucidating the influence of interspecies microbiota transplantation on plant growth

Through the pot experimentation it has also been noticed that the *G. hirsutum* plants transplanted with *G. arboreum* rhizospheric MFs had a higher growth rate compared to the relative negative control group. The growth rate has been reported in Supplementary Figure 23. This response was observed most significantly in rhizospheric transplantation groups of both PFV1 and PFV2 *G. hirsutum* (Supplementary Figure 23a and b) and was not found to be much pronounced in phyllospheric transplantations (Supplementary Figure 23c and d).

Supplementary results 3: DESeq2-Key differentiation in bacterial genera and their associated pathways in CLCuD-tolerant *G. arboreum* and variably susceptible *G. hirsutum*

Supplementary Figure 3 and 4 shows the comparative microbial diversity of Phyllospheric MFs. The Phyllosphere of *G. arboreum* FDH228 had several upregulated bacterial genera as compared to the Phyllosphere of *G. hirsutum* PFV1, including *Methylotenera, Methylophilus, Nevskia, Sinobaca, Reyranella, Empedobacter, Candidatus nomuravacteria, Methylobacillus* and many more (Supplementary Figure 3a). The Phyllospheric MF of the partially tolerant *G. hirsutum* variety PFV1 had many bacterial genera expressing significantly higher than that in FDH228 phyllosphere, including *Rhodococcus, Pseudonocardia, Sphingobacterium, Melittangium, Actinomycetospora, Marmoricola* and various others. However, the comparative analysis of FDH228 Phyllospheric MF and PFV2 Phyllospheric MF reveal only one genre upregulated in FDH228, which was *Sinobaca* (Supplementary Figure 3b), and four in PFV2, which were *Vogesella, Cronobacter, Escherichia-Shigella, Kosakonia* and *Pantoea*.

Supplementary results 4: Pathway Analysis as a basis of plant-microbe interactions and possible disease suppression

The comparative analysis of pathways in rhizosphere of FDH228 and PFV2 (Supplementary Figure 5 and 6) shows upregulation of two pathways in PFV2, namely L-

tryptophan biosynthesis and enterobacterial common antigen biosynthesis. Comparative analysis of Phyllospheric MFs for differentially expressed pathways revealed 8 pathways in comparison between FDH228 and PFV1 (Supplementary Figure 7a). Nylon-6 oligomer degradation and starch biosynthesis was found to be significantly upregulated in FDH228 phyllosphere. On the other hand, peptidoglycan biosynthesis V (β-lactam resistance), Mycolyl-arabinogalactan-peptidoglycan complex biosynthesis, Vitamin B6 Degradation, taurine degradation, phospholipases synthesis and N-acetylglucosamine, N-acetylmannosamine and N-acetylneuraminate degradation were found to be upregulated in PFV1. When compared to phyllosphere of PFV2, FDH228 had only one pathway upregulated, namely starch biosynthesis (Supplementary Figure 7b).

Supplementary results 5: Core microbiome signatures associated with CLCuD suppression in cotton microbiome transplants

The differential taxonomic coverage of core microbiome in the relative rhizospheric and phyllospheric fractions of the three varieties is elucidated in the form of heat trees in Supplementary Figure 10. As the node size indicates the number of the most unique OTUs, the comparison of all the heat trees reveals the rhizosphere of *G. arboreum* FDH228 as the most densely populated fraction, owing to the presence of a fairly larger number of nodes and branches. Several taxonomic branches can be seen to be selected in the rhizosphere of *G. arboreum,* unlike those of *G. hirsutum,* specifically the node of *Verrucomicrobiota* leading to *Lecunisphaera* and *Opitutus*. The branch starting with *Firmicutes* and leading to *Fictibacillus* is also among the diversely selected taxonomic levels by FDH228 rhizosphere. Other than those, several branches like *Alphaproteobacteria, Gammaproteobacteria* and *Patescibacteria,* leading to *Rhodospirillales,* Uncultured *Xanthobacteraeae* and *Saccharimonadales*, respectively, can be seen to be differentially expressed in rhizosphere of *G. arboreum* as compared to those of *G. hirsutum.*

Supplementary Discussion

Basis of natural CLCuD resistance in selected cotton varieties

When attacked by pathogens, plants undergo various intrinsic mechanisms at genetic level to overcome the biotic stress, including the release of elicitors which are in turn recognized by resistance genes in the host plant. The recognition of pathogen-associated molecular patterns (PAMPs) by the pattern recognition receptors (PRRs) in plant genome leads to the mobilization of signaling cascade and activation of pathogen triggered immunity (PTI) [6]. The natural resistance to biotic stresses attained by G. arboreum is also attributed to the evocation of general PAMP molecules and signal cascades that transport defense compounds throughout the plant [7]. A major reason relayed by past research for plants to acquire resistance to biotic stresses is the plant's microbiome. The plant-microbial association is increasingly seen as a reason behind elucidation of defense mechanisms in host plants [8]. The plant microbiome assembly is reported to be most affected by pathogen invasion [9]. The host plants can attract valuable microorganisms by the secretion of volatile organic compounds and specific root exudates [10], which devise keystone taxa in defending the plant against disease [11]. However, a few studies have highlighted the alteration of host plant's rhizospheric microbiome in response to pathogens attacking the aerial parts of the plant [12].

The cotton varieties used in the study have been selected based on their respective resistance levels to begomovirus, the causative agent for CLCuD. These plants were grown in uncontrolled conditions under natural photoperiod till their flowering stage, in the presence of viruliferous silverleaf white flies, (*Bemisia tabaci*), which were seen in large numbers under the cotton leaves (Supplementary Figure 12). Supplementary Figure 13 shows the developmental progress of leaves from G. hirsutum plant grown under controlled conditions and uncontrolled conditions. *G. hirsutum*, the tetraploid species, has been documented to be a result of polyploidization between the diploid species G. arboreum and *G. raimondii* [13]. Where G. hirsutum is susceptible to various biotic stresses including CLCuD, its ancestor G. arboreum has naturally attained resistance to several biotic and abiotic stresses [14]. Therefore, both the cotton species under begomovirus attack were chosen to elucidate the effect of their respective distinct microbiota on disease suppression. This elucidation of plant's microbiota in disease suppression remains the first insight into control of a viral plant disease and the first to be reported on cotton crop. While rhizospheric microbiome has gained much attention as a

way forward for sustainable agriculture, the phyllospheric microbiome remains ignored as a source eliciting plant defense response. The study, therefore, also proposes the novel disease suppressive role of plant phyllosphere.

Plant microbiome: the aide to plant in adverse conditions

Recent advances in next generation technology unveil the plant as a complete holobiont with each plant fostering billions of signature microorganisms involved in maintenance including germination, growth, defense, physiology, and productivity [15]. The diversity of environments the above and below ground parts of the plant interact with, has split the microbiome into two types: the phyllospheric microbiome or phyllobiome and rhizospheric microbiome or rhizobiome [16]. While the significance of rhizosphere has been extensively studied for the mediation of biotic stress management of the plant, the phyllosphere and its roles require deeper evaluation [17]. Governed by various biotic and abiotic environmental factors, a plant's microbiome assembly varies depending on the plant compartment inhabited (e.g., rhizosphere, root endosphere, leaf episphere or leaf endosphere) [18], climatic changes [19], soil type [20], and pathogen invasion [21]. Studies on wheat [22], sugar beet [23] and *Arabidopsis thaliana* [12] establish the fact that the roots of pathogen-infected plants anchor beneficial microbial candidates in their microbiota to help rescue the plant during pathogen infection. This "cry for help" strategy imparts a higher diversity to rhizospheric and phyllospheric microbiota of plants under pathogen attack. The plant's "cry" refers to the exudation of volatile organic compounds (VOCs) and other various root exudates which call beneficial microorganisms into the 3mm soil-root interaction layer of rhizospheric region to the diseased plant's aid [24]. The keystone microbial taxa constituting a diseased plant's microbiome might interact with the plant to suppress the incoming infection by secretion of antibiotic compounds, enhancing competition of resources with the pathogen or by priming the plant's immune system [11].

Building on these crucial findings, this study aims to develop the plant's own microbiome as a potential candidate in inducing disease suppression. Various studies reported earlier using plant microbiome for disease suppression have dealt with bacterial or fungal plant diseases, but this study is an effort to analyze the effect of plant microbiota on the suppression of a viral plant disease. As cotton is the 4th largest cash crop of Pakistan,

the strategy is aimed at preventing the crashing economy of the crop using agro-economic means in sustainable agriculture and bring cotton back to its decades' old vigor. CLCuD caused by begomovirus is one of the most detrimental threats to the crop with devastating losses each year. As indicated in various recent studies, the rhizospheric community has been reported to be altered by the above ground pathogens, resulting in recruitment of a higher concentration of phytohormones JA, SA and ABA in the shoot to help plant combat the pathogen attack [25]. The findings also indicate that the members of plant microbiota confer extension in the immune functions of the host plant [26]. Strong evidence suggests the activation of microbiota-modulated immunity in plants, referred to as MMI, enhances the ability of the host plant to resist disease [27]. Since a healthy plant is actively colonized by commensal microorganisms, and the pattern recognition receptors (PRRs) are unable to discriminate commensal microbes from pathogenic ones, it is suggested that a medium of instruction exists between the microbiota and the host plant, laying the basis of communication beyond the basic coevolutionary arms race [28].

Supplementary Tables

Supplementary Table 1: Test Group Codes and Microbial Fraction Application details.

**Test Group Code**		**Detail**
**PFV1.RMF**	Rhi.RMF	*Gossypium hirsutum* PFV1 plants to which Microbial Fraction from FDH228 *arboreum's* **Rhi**zosphere has been applied
	Phy.RMF	*Gossypium hirsutum* PFV1 plants to which Microbial Fraction from FDH228 *arboreum's* **Phy**llosphere has been applied
**PFV1.pRMF**	Rhi.pRMF	*Gossypium hirsutum* PFV1 plants to which Microbial Fraction from PFV1 *hirsutum's* **Rhi**zosphere has been applied
	Phy.pRMF	*Gossypium hirsutum* PFV1 plants to which Microbial Fraction from PFV1 *hirsutum's* **Phy**llosphere has been applied
**PFV1.SMF**	Rhi.SMF	*Gossypium hirsutum* PFV1 plants to which Microbial Fraction from PFV2 *hirsutum's* **Rhi**zosphere has been applied
	Phy.SMF	*Gossypium hirsutum* PFV1 plants to which Microbial Fraction from PFV2 *hirsutum's* **Phy**llosphere has been applied
**PFV1.SA**	N/A	*Gossypium hirsutum* PFV1 plants to which **S**alicylic **A**cid has been applied
**PFV1.nMF**	N/A	*Gossypium hirsutum* PFV1 plants to which **n**o **M**icrobial **F**raction has been applied
**PFV2.RMF**	**Rhi.RMF**	*Gossypium hirsutum* PFV2 plants to which Microbial Fraction from FDH228 *arboreum's* **Rhi**zosphere has been applied
	Phy.RMF	*Gossypium hirsutum* PFV2 plants to which Microbial Fraction from FDH228 *arboreum's* **Phy**llosphere has been applied

**PFV2.pRMF**	Rhi.pRMF	*Gossypium hirsutum* PFV2 plants to which Microbial Fraction from PFV1 *hirsutum's* **Rhi**zosphere has been applied
	Phy.pRMF	*Gossypium hirsutum* PFV2 plants to which Microbial Fraction from PFV1 *hirsutum's* **Phy**llosphere has been applied
**PFV2.SMF**	Rhi.SMF	*Gossypium hirsutum* PFV2 plants to which Microbial Fraction from PFV2 *hirsutum's* **Rhi**zosphere has been applied
	Phy.SMF	*Gossypium hirsutum* PFV2 plants to which Microbial Fraction from PFV2 *hirsutum's* **Phy**llosphere has been applied
**PFV2.SA**	N/A	*Gossypium hirsutum* PFV2 plants to which **S**alicylic **A**cid has been applied
**PFV2.nMF**	N/A	*Gossypium hirsutum* PFV2 plants to which **n**o **M**icrobial **F**raction has been applied
**FDH228.RMF**	**Rhi.RMF**	*Gossypium arboreum* FDH228 plants to which Microbial Fraction **from FDH228 *arboreum's* Rhizosphere has been applied**
	Phy.RMF	*Gossypium arboreum* FDH228 plants to which Microbial Fraction from FDH228 *arboreum's* **Phy**llosphere has been applied
**FDH228.pRMF**	Rhi.pRMF	*Gossypium arboreum* FDH228 plants to which Microbial Fraction from PFV1 *hirsutum's* **Rhi**zosphere has been applied
	Phy.pRMF	*Gossypium arboreum* FDH228 plants to which Microbial Fraction from PFV1 *hirsutum's* **Phy**llosphere has been applied
**FDH228.SMF**	Rhi.SMF	*Gossypium arboreum* FDH228 plants to which Microbial Fraction from PFV2 *hirsutum's* **Rhi**zosphere has been applied

	Phy.SMF	*Gossypium arboreum* FDH228 plants to which Microbial Fraction from PFV2 *hirsutum's* **Phy**llosphere has been applied
**FDH228.SA**	N/A	*Gossypium arboreum* FDH228 plants to which **S**alicylic **A**cid has been applied
**FDH228.nMF**	N/A	*Gossypium arboreum* FDH228 plants to which **n**o **M**icrobial **F**raction has been applied

Supplementary Table 2: Primers used for *Begomovirus* DNA amplification

(IUB codes for degenerate bases: M=A/C, R=A/G, W=A/T, S=G/C, Y=C/T. K=G/T,V=A/G/C, H=A/C/T, D=A/G/T, B=G/C/T, N=A/G/C/T

Primer Code	***Begomovirus*** **DNA region amplified**	Company	Primer sequence (5'-3')
**Beta1(Forward)**	β-satellite	Synbio Tech	GGTACCACTACGCTACGCAGCAGCC
**Beta2 (Reverse)**	β-satellite	Synbio Tech	GGTACCTACCCTCCCAGGGGTACAC

Supplementary Table 3: Polymerase chain reaction profile for β-satellite amplification (Thermocycler: Nyx Technik, Inc. Model: A4)

Step	Name	Temp/ºC	Time/min	
**1**	Denaturation	94	4	
**2**	Denaturation	94	1	30 Cycles
**3**	Annealing	68	1	
**4**	Extension	72	1.5	
**6**	Final Extension	72	10	
**7**	Cooling	5	5	

Supplementary Table 4: Raw data statistics for RNA samples

Sample ID	Total bases (bp)	Total reads	GC (%)	AT (%)	Q20 (%)	Q30 (%)
PFV1_Rhi_RMF1	1,393,563,598	9,228,898	54.7	45.3	96.1	93.2
PFV1_Rhi_RMF2	1,427,923,044	9,456,444	50.8	49.2	97.6	95.0
PFV1_Rhi_RMF3	390,020,920	2,582,920	51.8	48.2	96.4	93.5
PFV1_Rhi_SMF1	1,283,532,918	8,500,218	55.7	44.3	95.5	92.4
PFV1_Rhi_SMF2	1,321,338,788	8,750,588	56.0	44.0	94.7	89.6
PFV1_Rhi_nMF1	1,344,657,718	8,905,018	51.4	48.6	97.0	92.4
PFV1_Rhi_nMF2	1,896,602,280	12,560,280	41.5	58.5	87.2	79.4

Supplementary Table 5: Transcriptomics summary statistics

Sample	Total reads after quality trimming	Total mapped reads to the reference
nMF1	26797490	5998365
nMF2	14419477	6057355
RMF1	31439591	5710981
RMF2	23751277	5370656
RMF3	7577932	1685037
SMF1	27861527	4864974
SMF3	31705686	5546838

Supplementary Figures

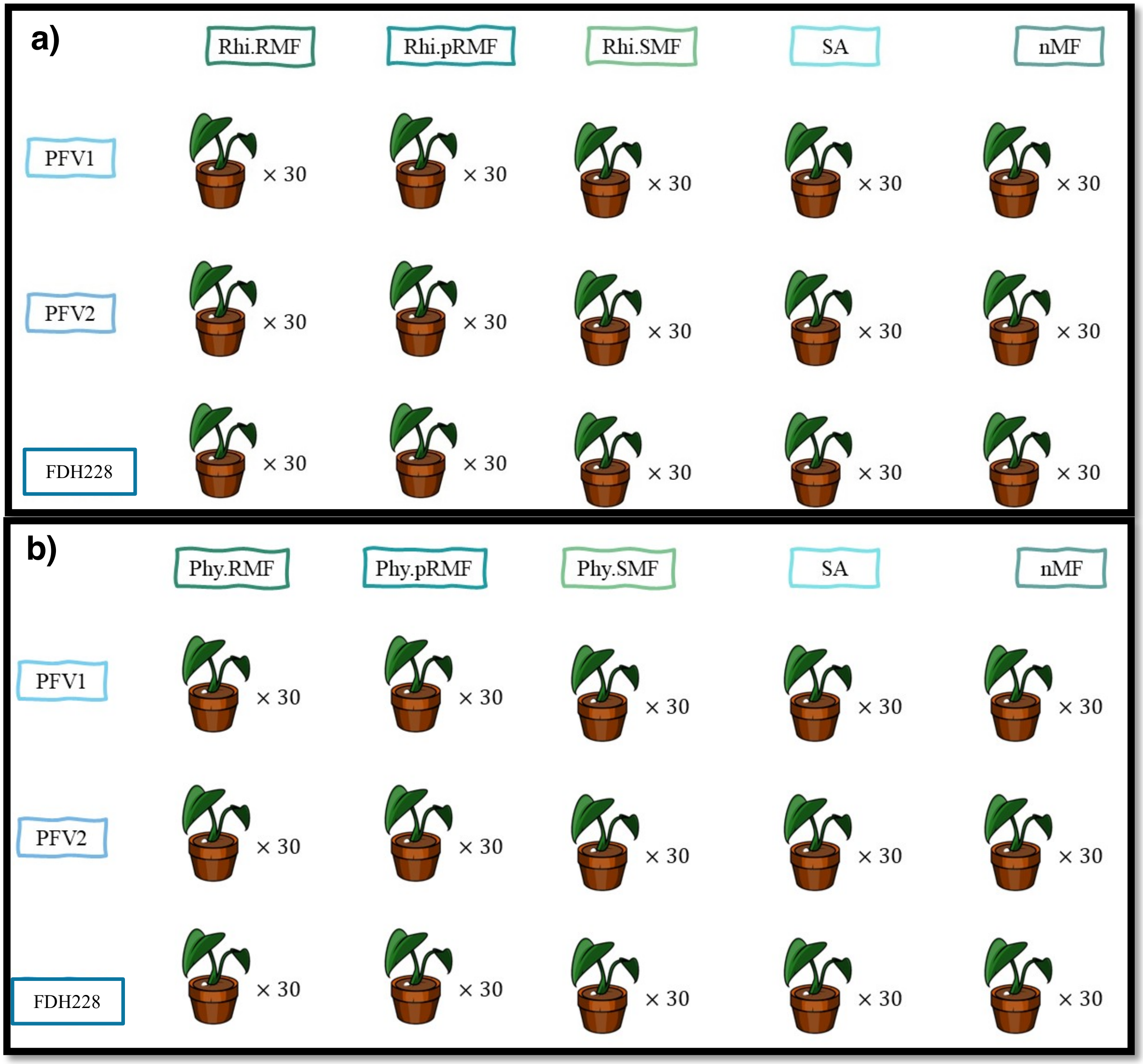

Supplementary Figure 1. Microbial Fraction application plan. **a)** Application test groups for Rhizospheric Microbial Fractions. **b)** Application test groups for Phyllospheric Microbial Fractions

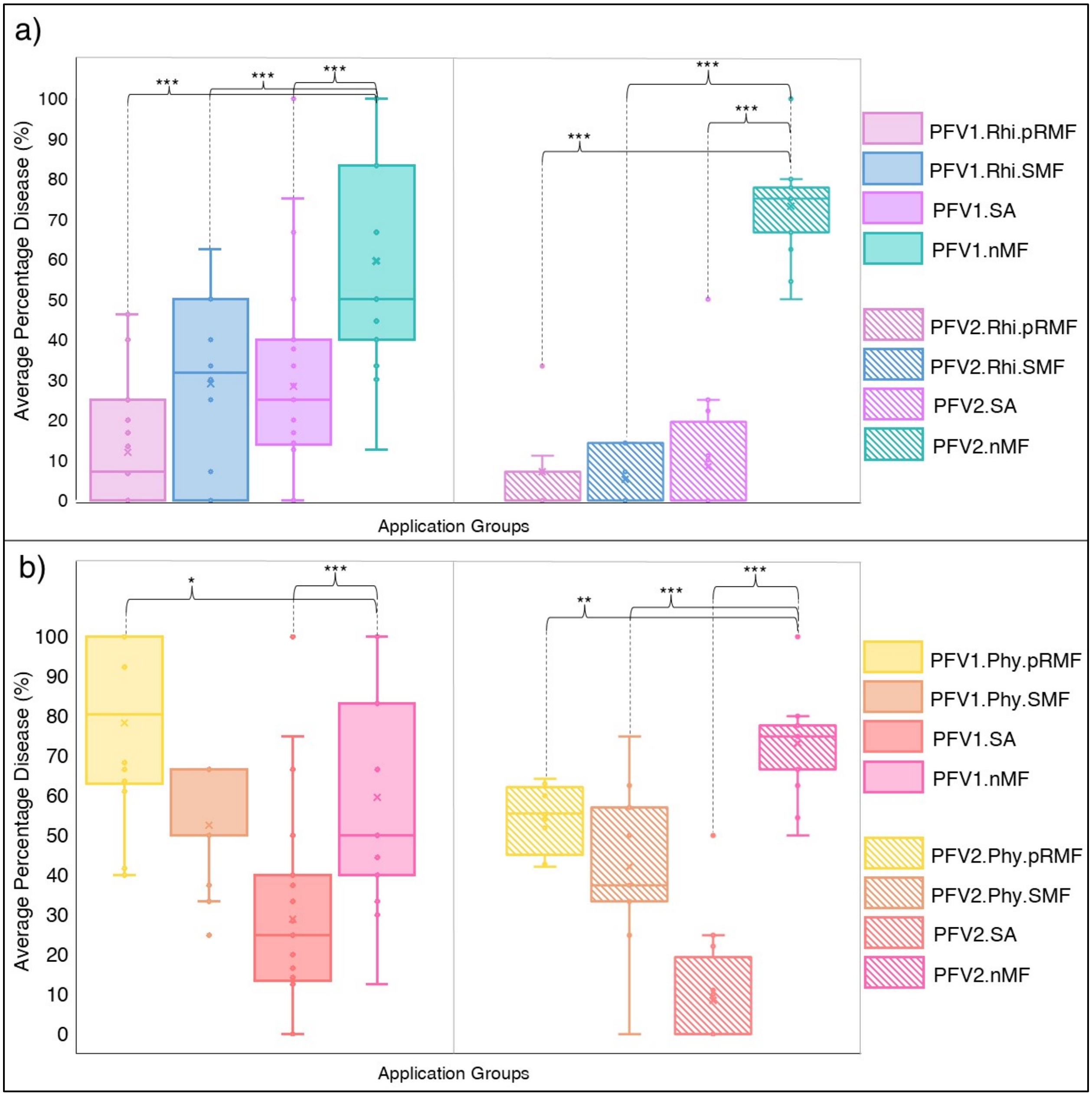

Supplementary Figure 2. CLCuD progress for 60th day-post-inoculation in cotton varieties treated with Rhizospheric and Phyllospheric Microbial Fractions and exogenous Salicylic Acid. a) CLCuD progress in *G. hirsutum* partially tolerant and completely tolerant varieties, PFV1 and PFV2, under Rhi.MFs application. **b)** CLCuD progress in *G. hirsutum* partially tolerant and completely tolerant varieties, PFV1 and PFV2, under Phy.MFs application.

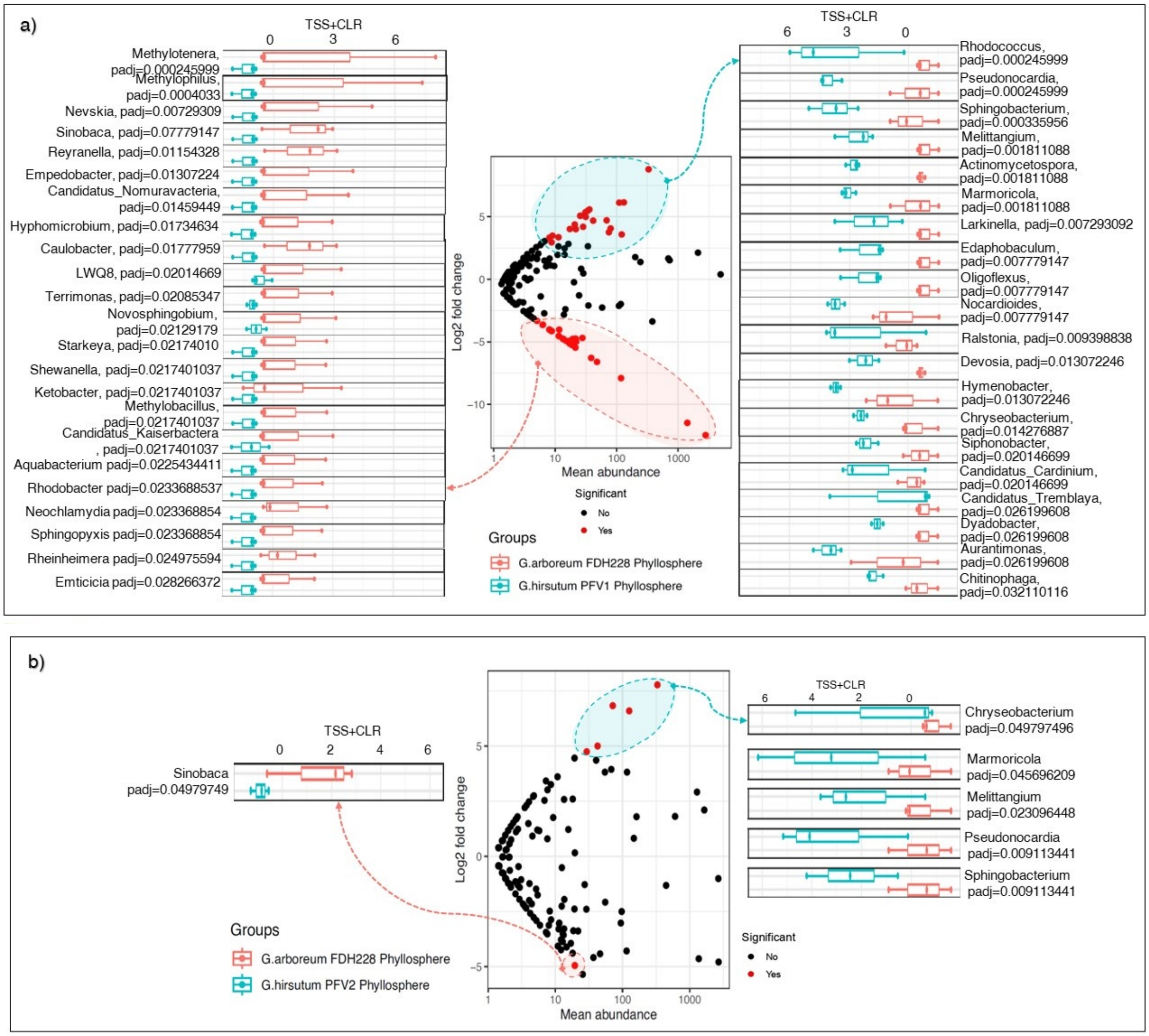

Supplementary Figure 3. Comparative analysis of Phy.MFs from *G. arboreum* FDH228 and *G. hirsutum* PFV1 and PFV2 using DESeq2. a) Log2Fold change in bacterial genera plotted against mean abundance comparing FDH228 rhizospheric MF and PFV1 Phyllospheric MF, and key genera with significantly higher abundance in FDH228 Phyllospheric MF than in PFV1 Phyllospheric MF. **b)** Log2Fold change in bacterial genera plotted against mean abundance comparing FDH228 Phyllospheric MF and PFV2 Phyllospheric MF, and key genera with significantly higher abundance in FDH228 Phyllospheric MF than in PFV2 Phyllospheric MF.

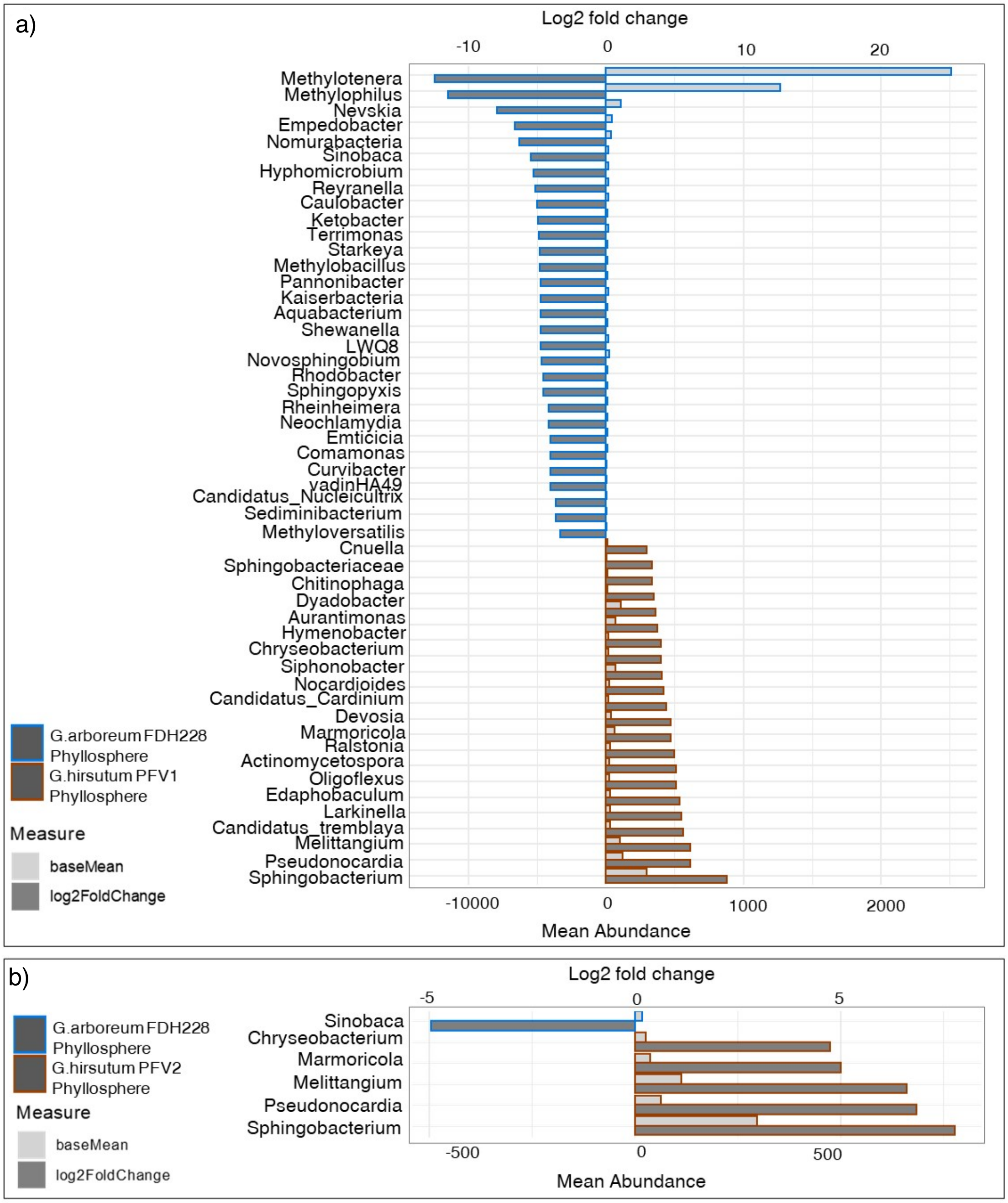

Supplementary Figure 4. Comparison of bacterial genera selected in Phy.MFs from *G. arboreum* FDH228 and *G. hirsutum* PFV1 and PFV2. a) Key bacterial genera selected in FDH228 and PFV1 Phyllosphere. **b)** Key bacterial genera selected in FDH228 and PFV2 Phyllosphere.

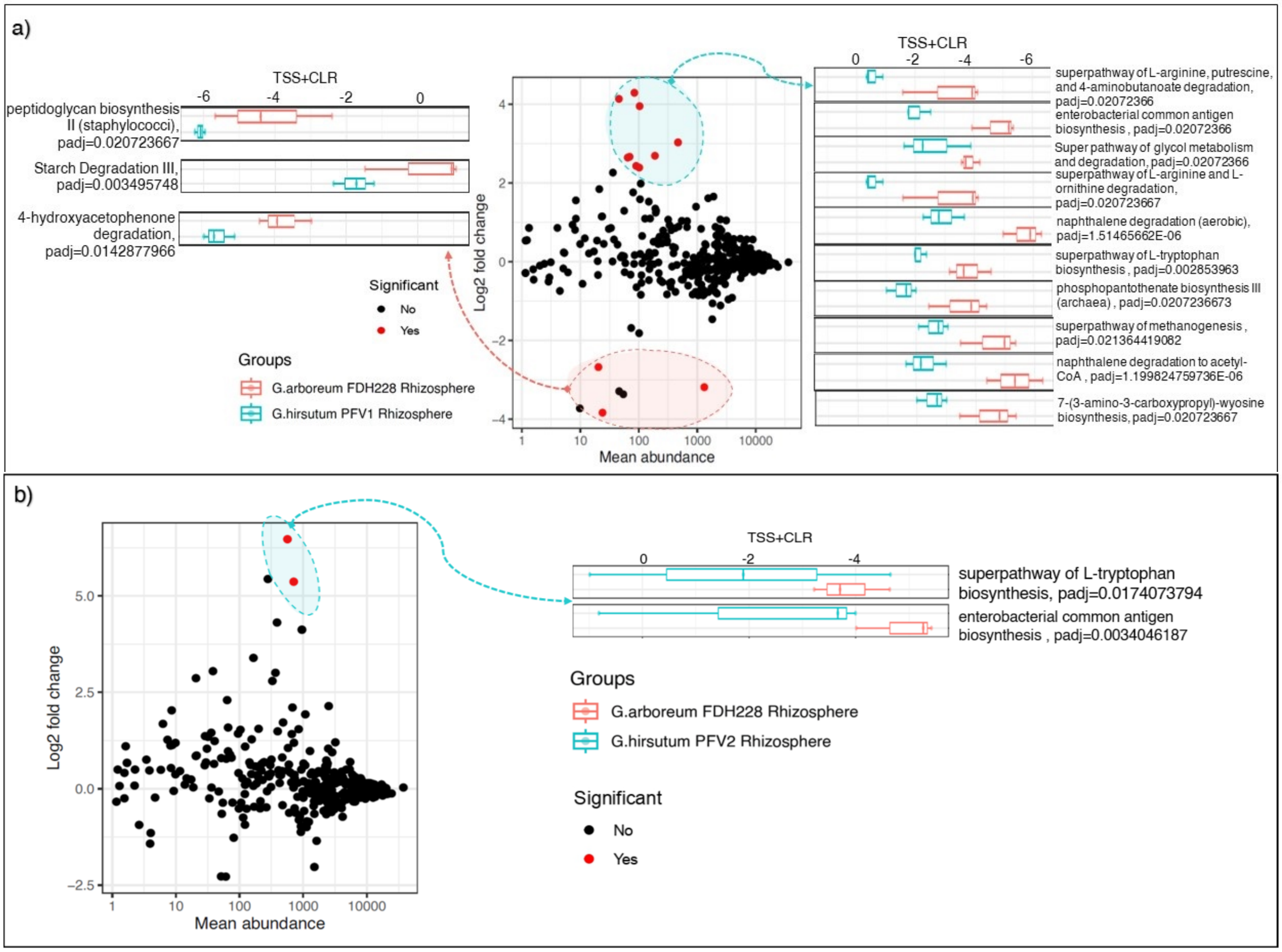

Supplementary Figure 5. Comparative analysis of metabolic pathways in Rhi.MFs from *G. arboreum* FDH228 and *G. hirsutum* PFV1, PFV2 using DESeq2. a) Log2Fold change in pathways plotted against mean abundance comparing FDH228 rhizospheric pathways and PFV1 Rhizospheric pathways. **b)** Log2Fold change in pathways plotted against mean abundance comparing FDH228 rhizospheric pathways and PFV1 Rhizospheric pathways.

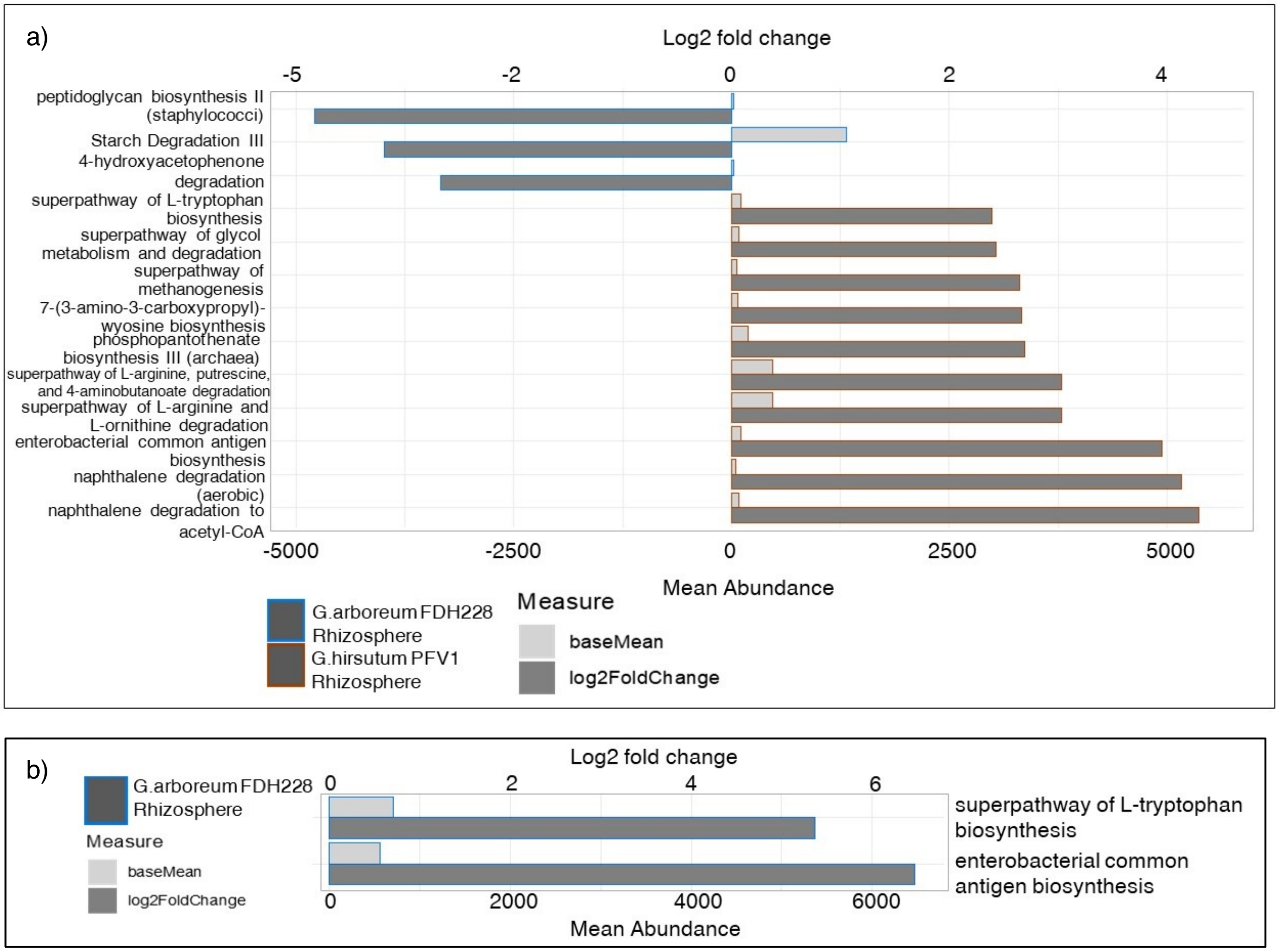

Supplementary Figure 6. **Metabolic pathways selected in Rhi.MFs from *G. arboreum* FDH228 and *G. hirsutum* PFV1, PFV2. a)** Key metabolic pathways selected in FDH228 and PFV1 Rhizosphere. **b)** Key metabolic pathways selected in FDH228 and PFV2 Rhizosphere.

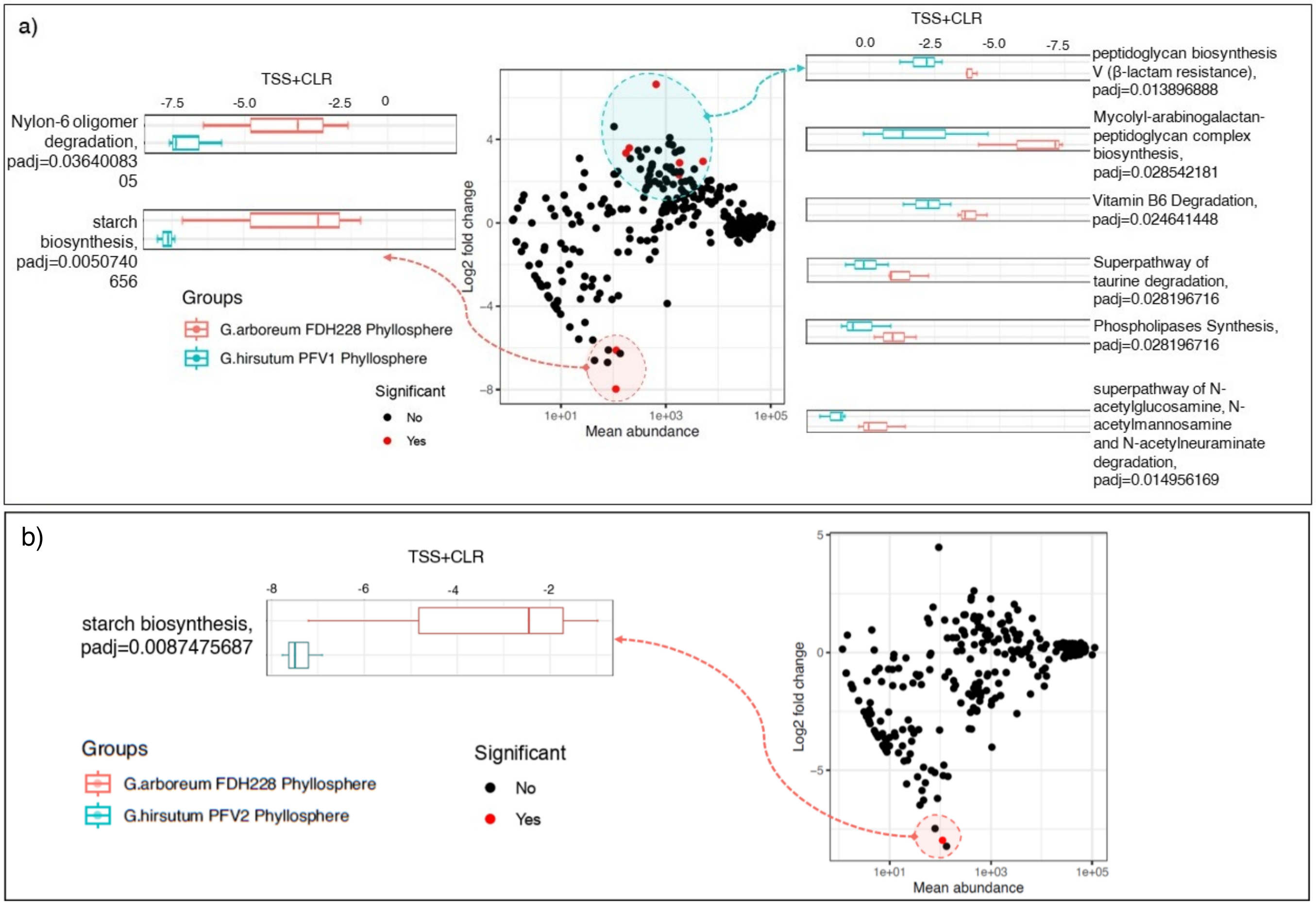

Supplementary Figure 7. Comparative analysis of metabolic pathways in Phy.MFs from *G. arboreum* FDH228 and *G. hirsutum* PFV1, PFV2 using DESeq2. a) Log2Fold change in pathways plotted against mean abundance comparing FDH228 phyllospheric pathways and PFV1 phyllospheric pathways. **b)** Log2Fold change in pathways plotted against mean abundance comparing FDH228 phyllospheric pathways and PFV1 phyllospheric pathways.

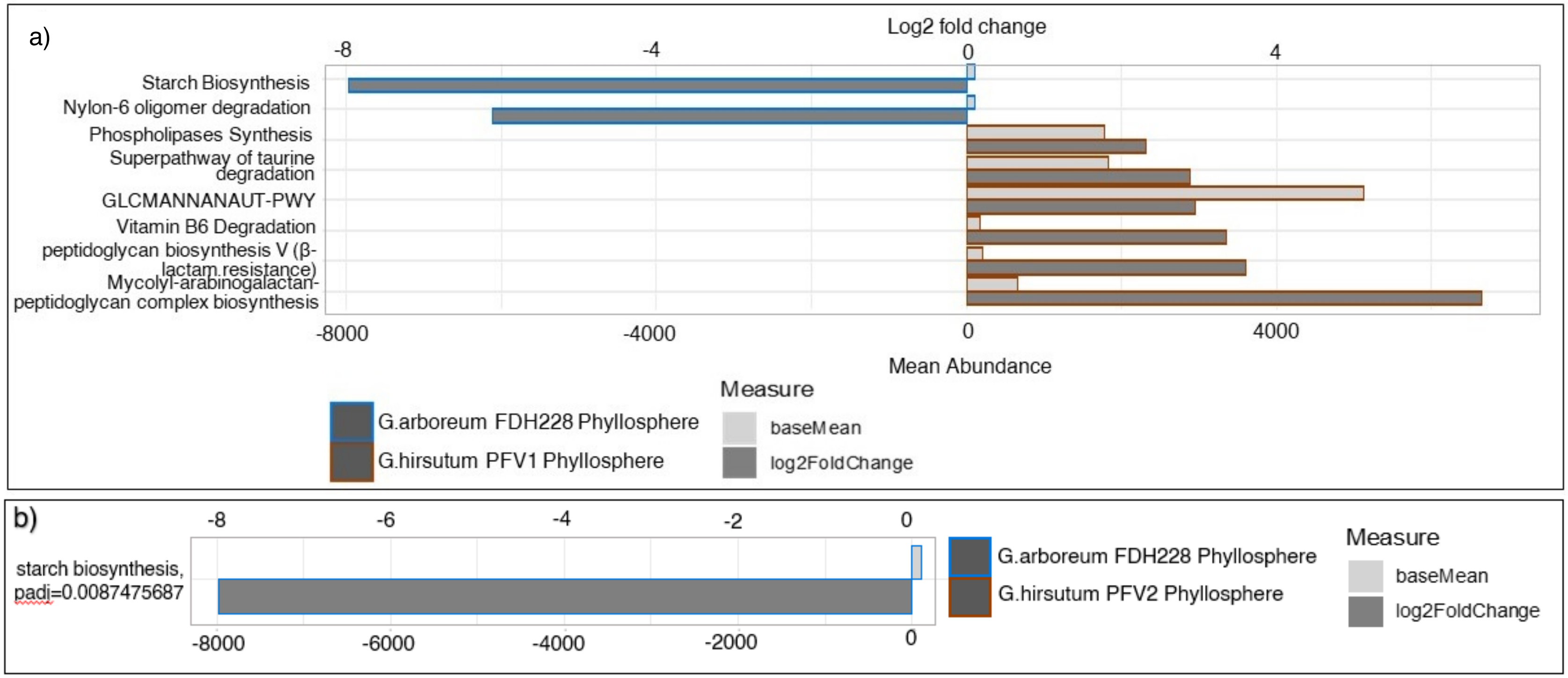

Supplementary Figure 8. **Metabolic pathways selected in Phy.MFs from *G. arboreum* FDH228 and *G. hirsutum* PFV, PFV2** **a)** Key downregulated and upregulated pathways in FDH228 phyllospheric MF and PFV1 phyllospheric MF. **b)** Key downregulated and upregulated pathways in FDH228 phyllospheric MF and PFV2 phyllospheric MF.

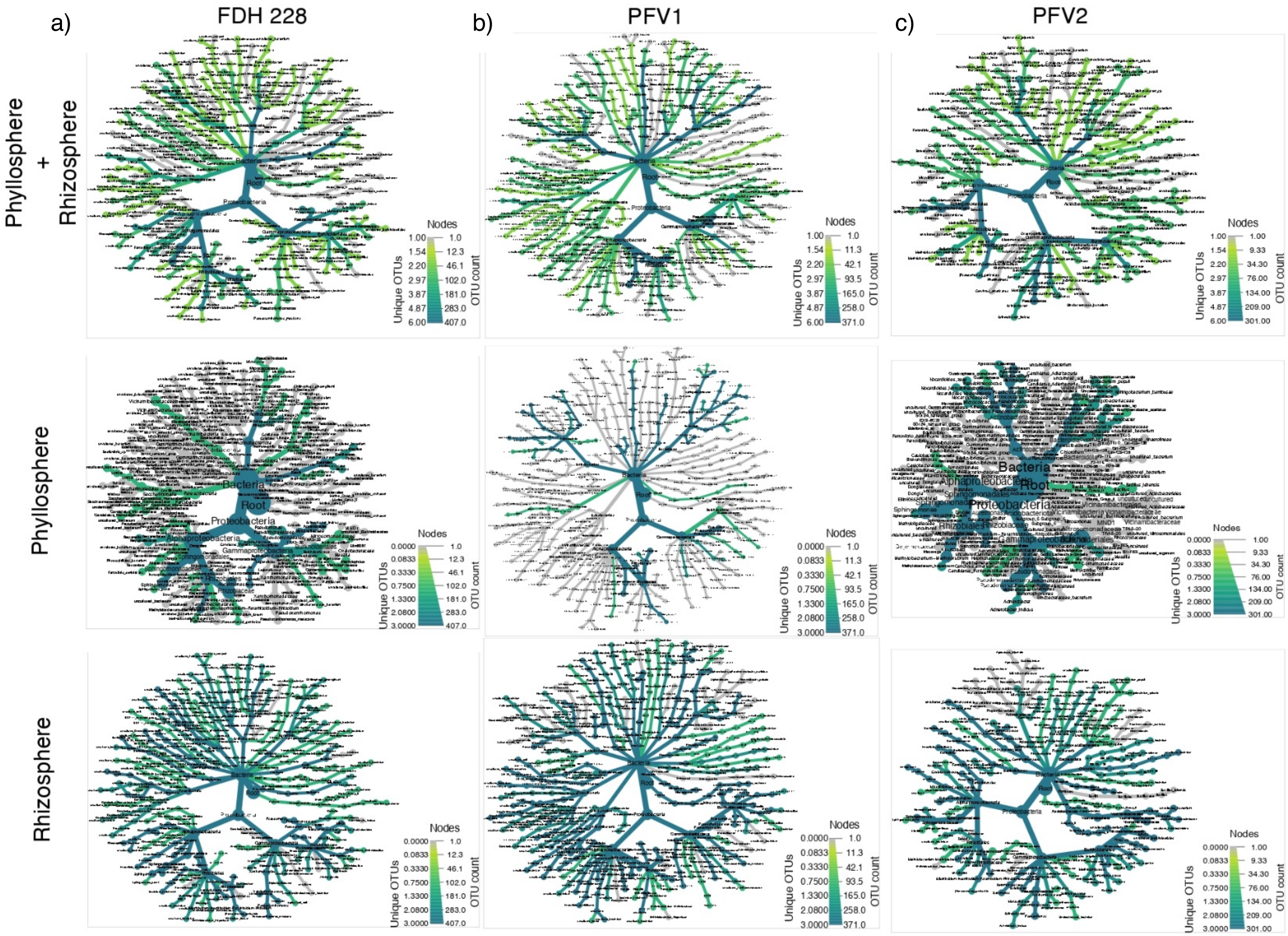

Supplementary Figure 9. Taxonomic coverage of core microbiome in (a) FDH 228, **(b)** PFV1, and **(c)** PFV2 as calculated in Figure 5. Top row collates abundances from all occupancies, shown separately as Phyllosphere (middle row) and Rhizosphere (bottom row). The width of the accompanying key is node size showing the number of unique OTUs (values on the left) whilst the colour is the abundance of these OTUs (values on the right).

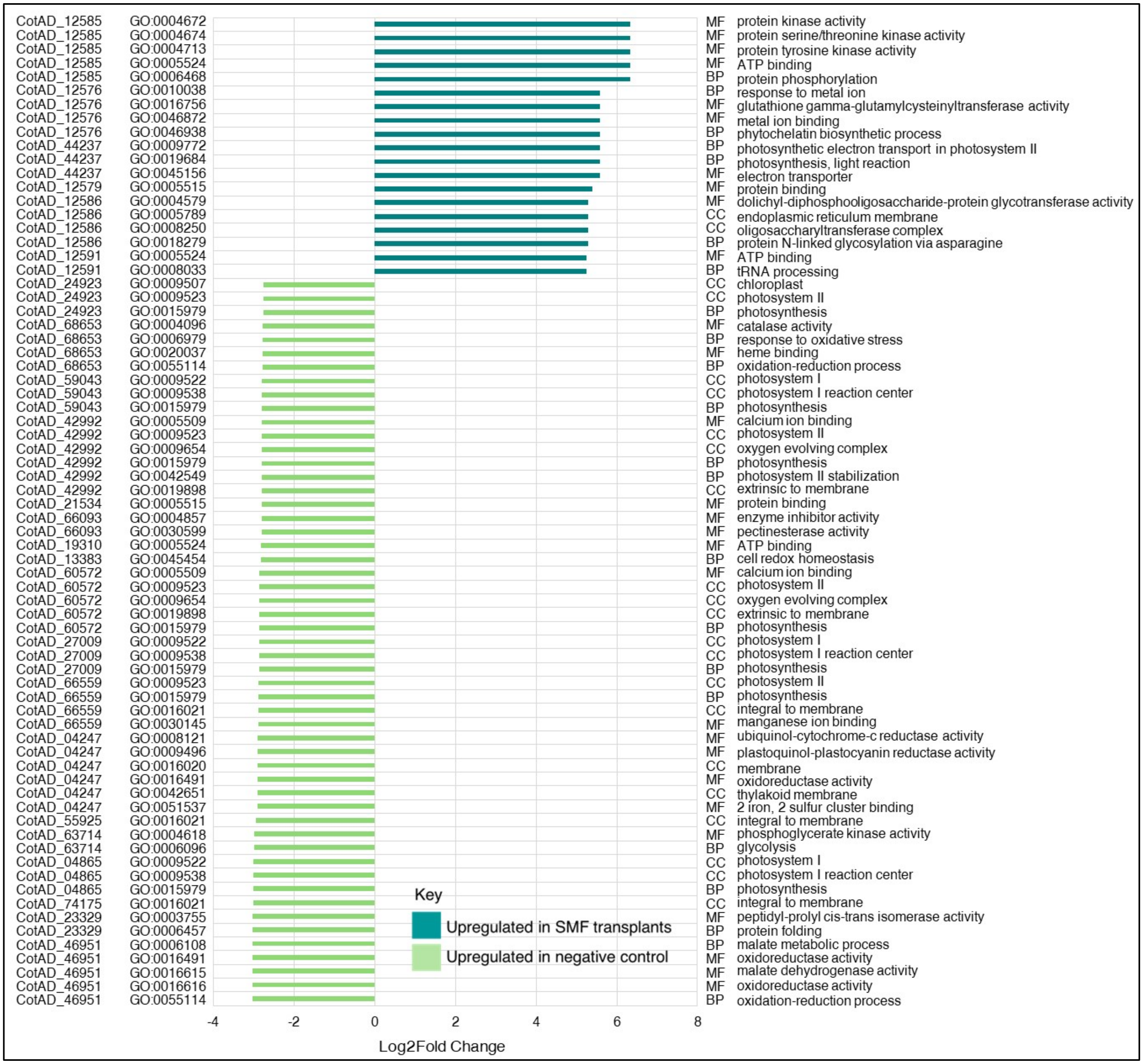

Supplementary Figure 10. Log2fold change in gene expression for hypothesis 2, nMF vs SMF. Log2fold change in genes upregulated in negative control and SMF transplants accompanied by original gene IDs, GO IDs, gene ontologies and functional annotations.

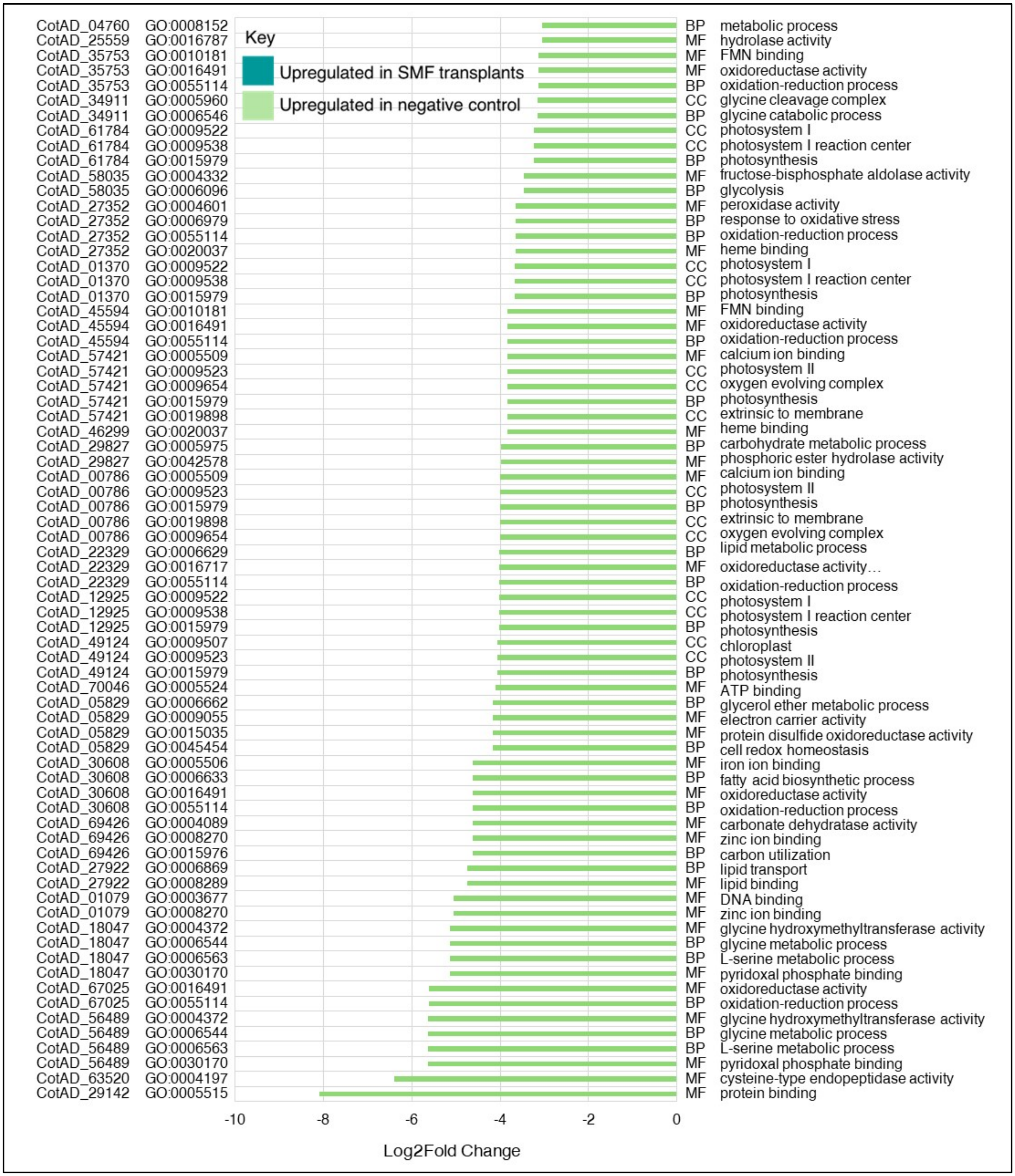

Supplementary Figure 11. Log2fold change in gene expression for hypothesis 2, nMF vs SMF. Continuation of Supplementary Figure 11.

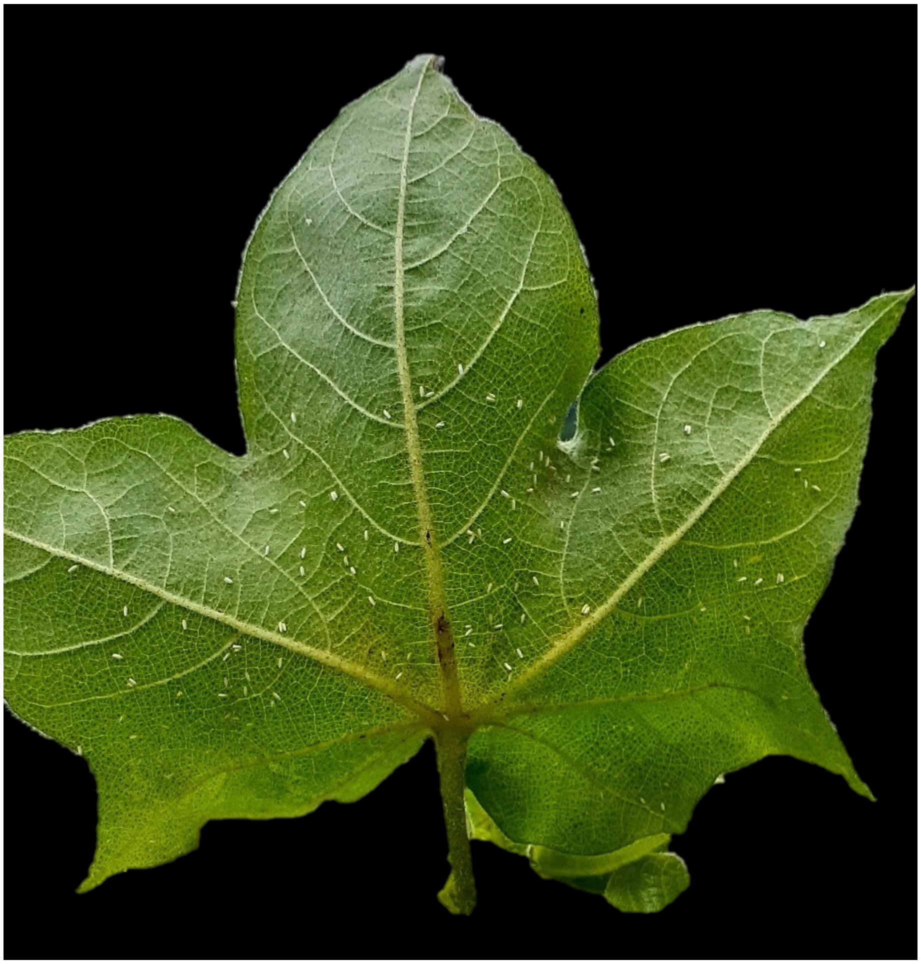

Supplementary Figure 12. The lower side of *Gossypium hirsutum* leaf with whitefly attack.

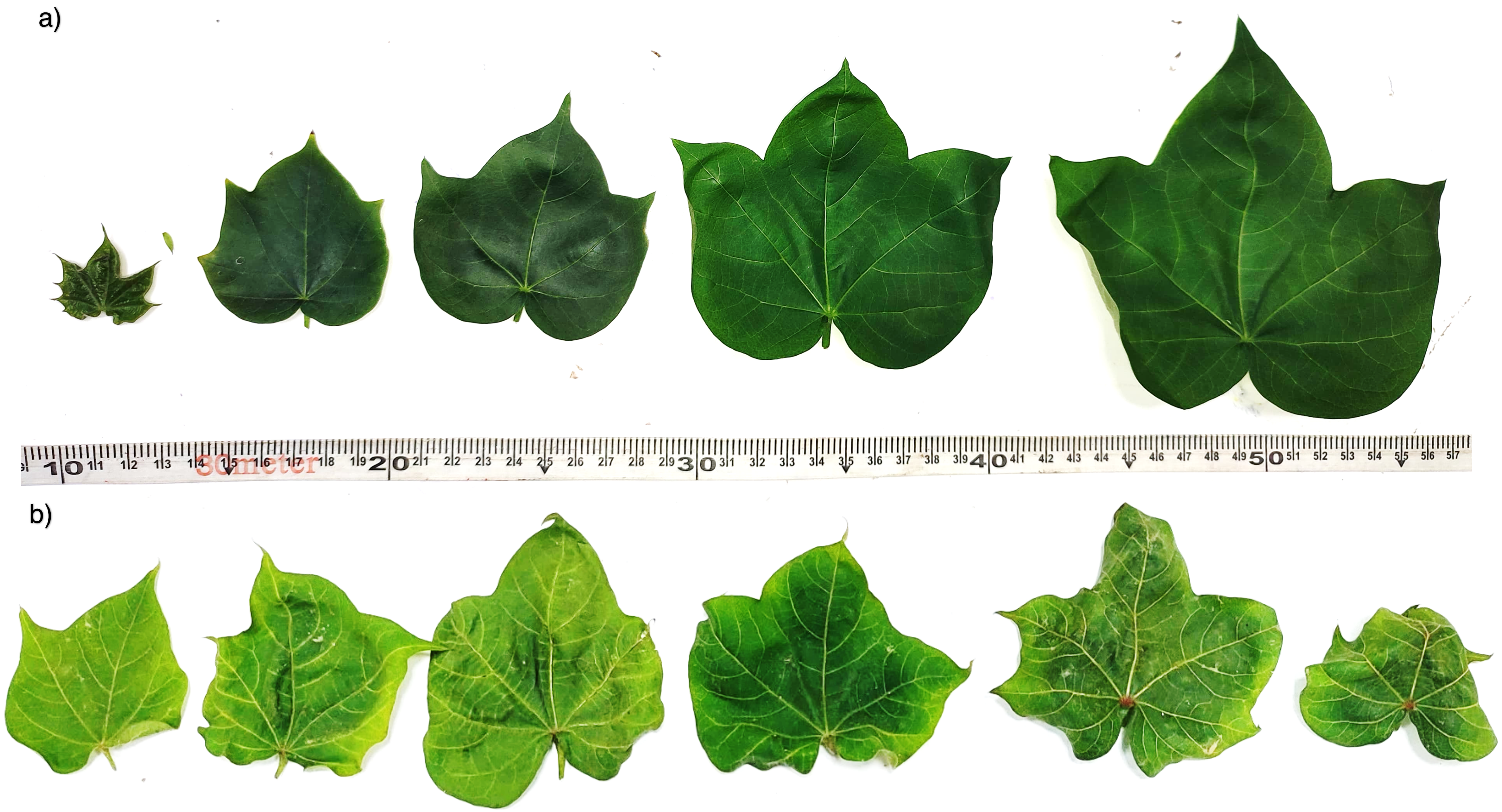

Supplementary Figure 13. *G. hirsutum* leaves in the stages of development. **a)** Leaves from a *G. hirsutum* plant grown in controlled conditions under 18 hours of light and 6 hours of dark, without viruliferous whitefly attack, **b)** Leaves from a *G. hirsutum* plant grown in uncontrolled conditions under natural photoperiod in the hub of viruliferous whitefly, depicting ideal curling symptoms of CLCuD

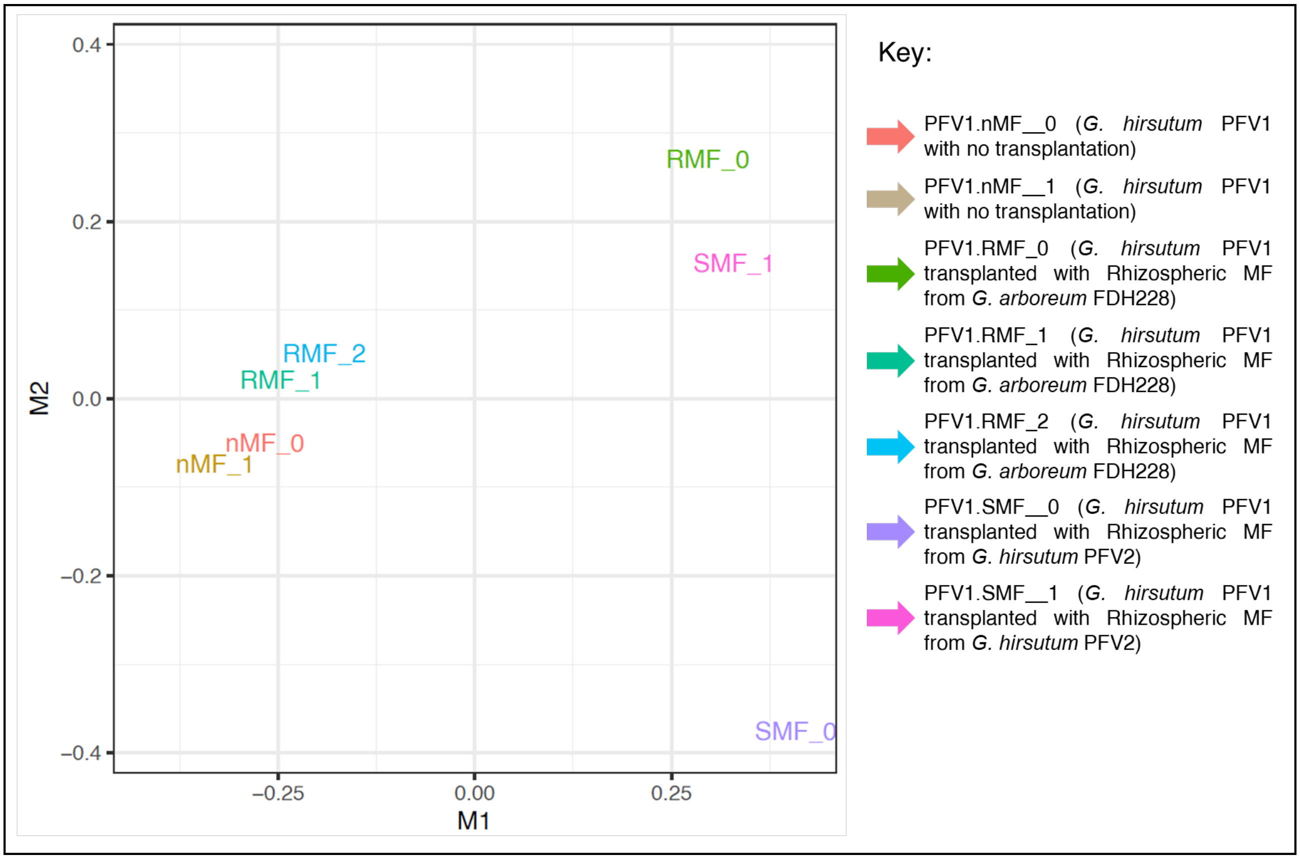

Supplementary Figure 14. Multidimensional Scaling (MDS) Plot of RNA-Seq Data Samples. Similarity and variability among different RNA-Seq data samples is shown. Each point represents an individual sample, and the distances between points reflect the dissimilarities in their gene expression profiles. The closer the points are, the more similar the samples. The plot highlights the clustering of samples based on their expression patterns, indicating potential biological differences or similarities among the groups.

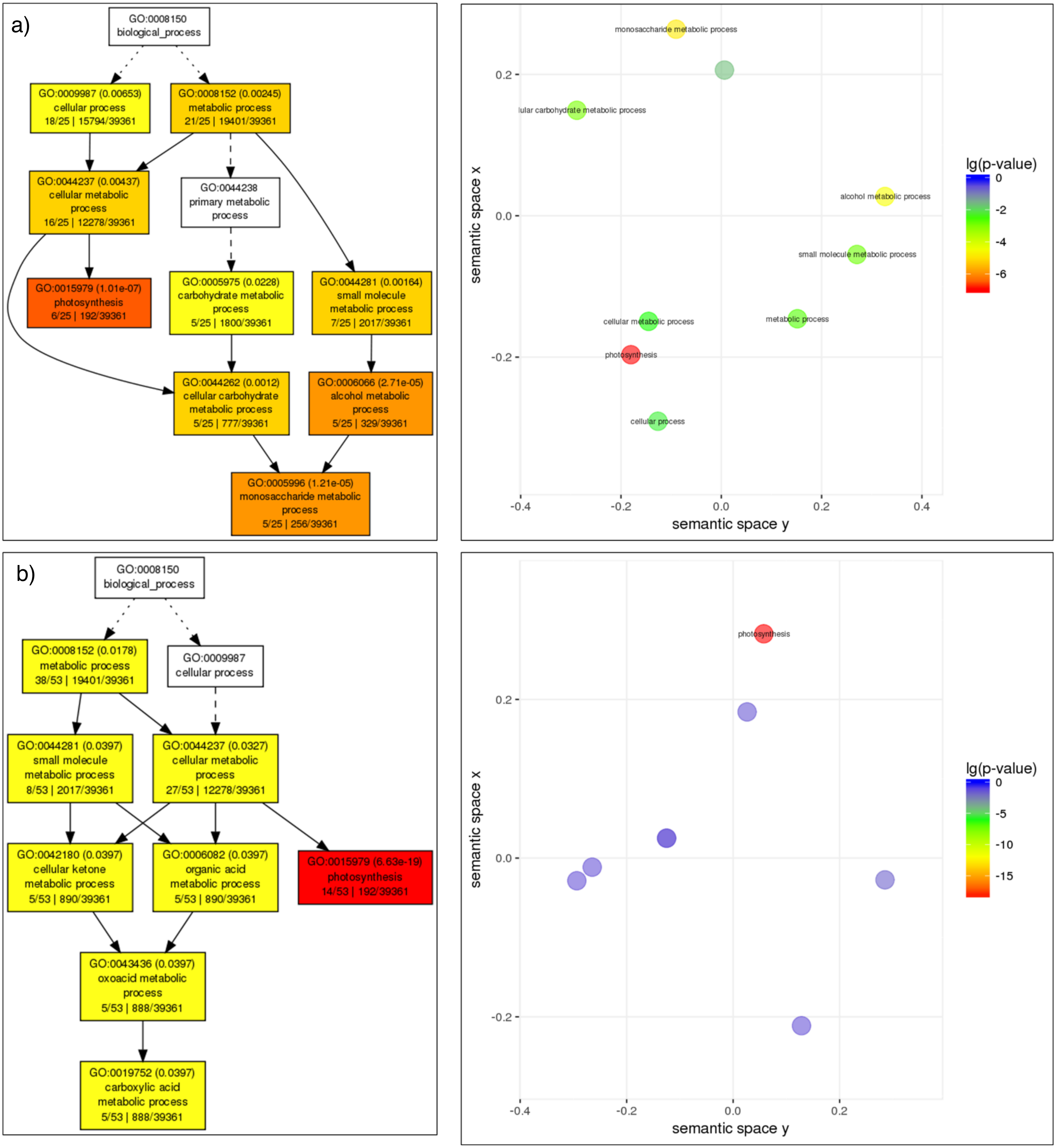

Supplementary Figure 15. Singular Enrichment Analysis (SEA) profiles for Hypothesis 1, nMF vs RMF, and Hypothesis 2, nMF vs SMF, obtained from AgriGo.v2. a) SEA profile for hypothesis 1 for Biological Process. **b)** SEA profile for hypothesis 2 for Biological Process.

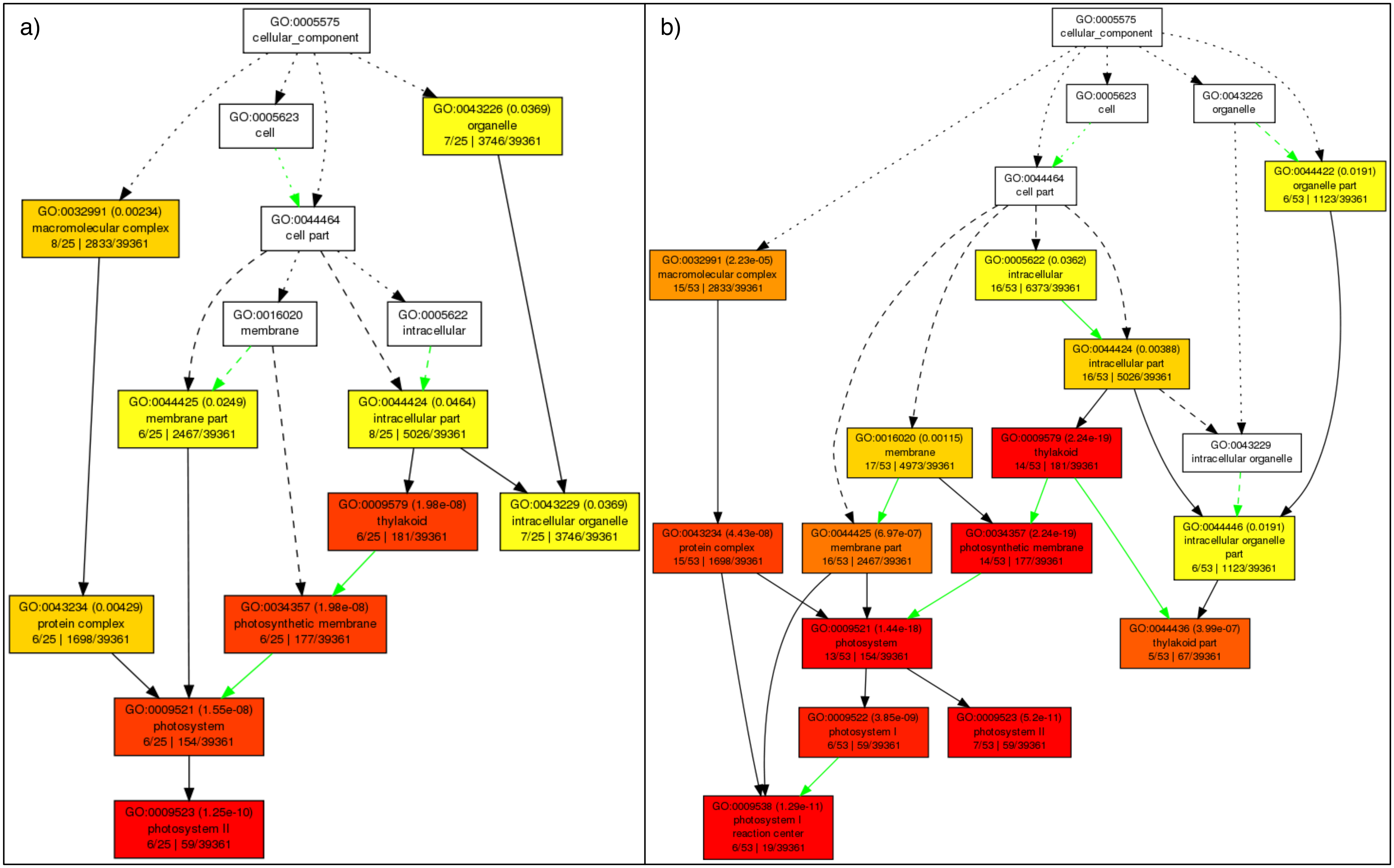

Supplementary Figure 16. Singular Enrichment Analysis (SEA) profiles for Hypothesis 1, nMF vs RMF, and Hypothesis 2, nMF vs SMF, obtained from AgriGo.v2. a) SEA profile for hypothesis 1 for Cellular Component. **b)** SEA profile for hypothesis 2 for Cellular Component.

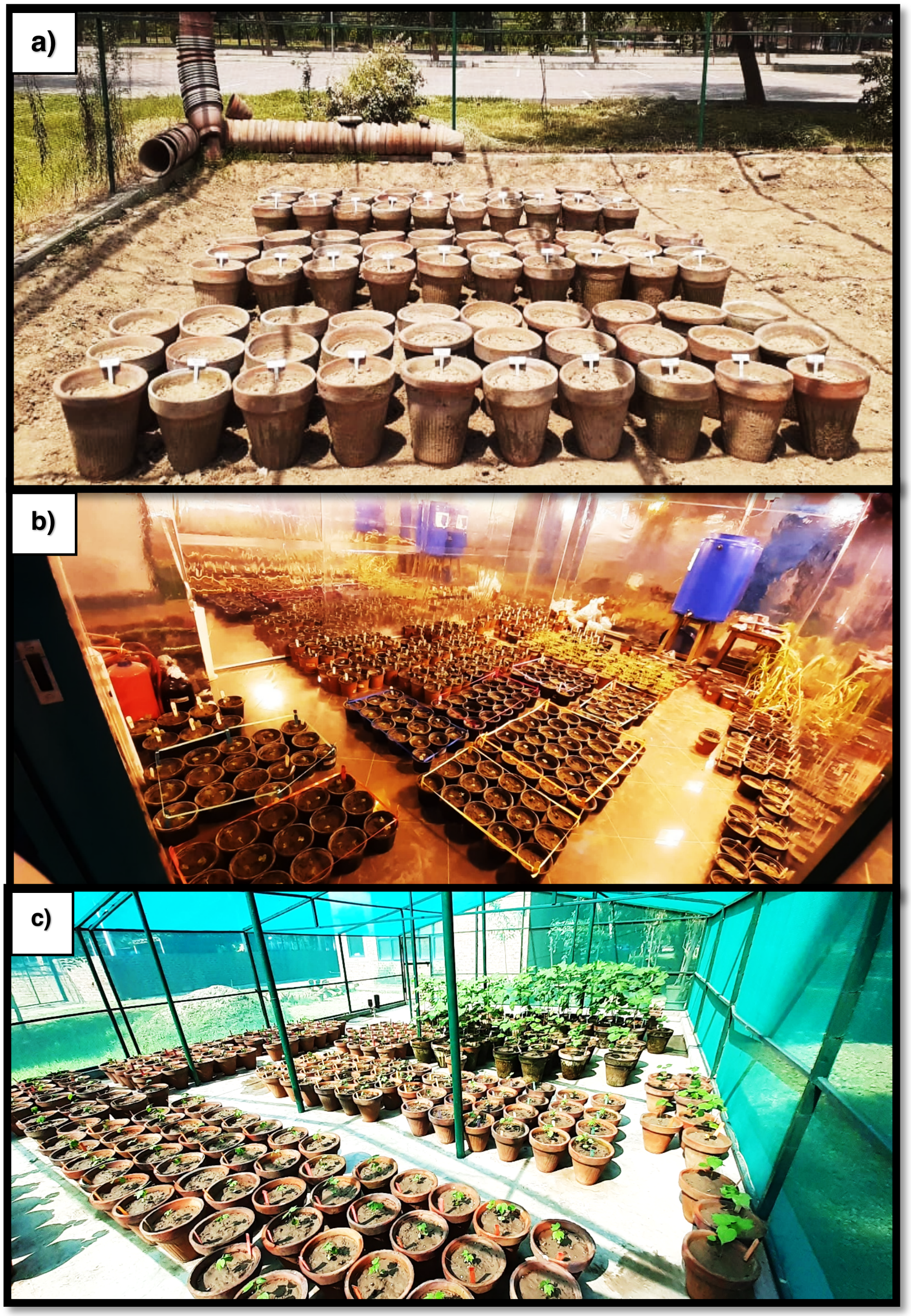

Supplementary Figure 17. Pot experiment conditions and set up. **a)** Net house I having uncontrolled conditions and reared viruliferous whitefly with natural photoperiod, reserved for plant batches for MF isolation. **b)** Climate-controlled room for germination and 4 weeks of pest free environment with an artificially maintained photoperiod of 16 hours of light, 8 hours of dark and 25°C. **c)** Net house II having uncontrolled conditions and reared viruliferous whitefly with natural photoperiod.

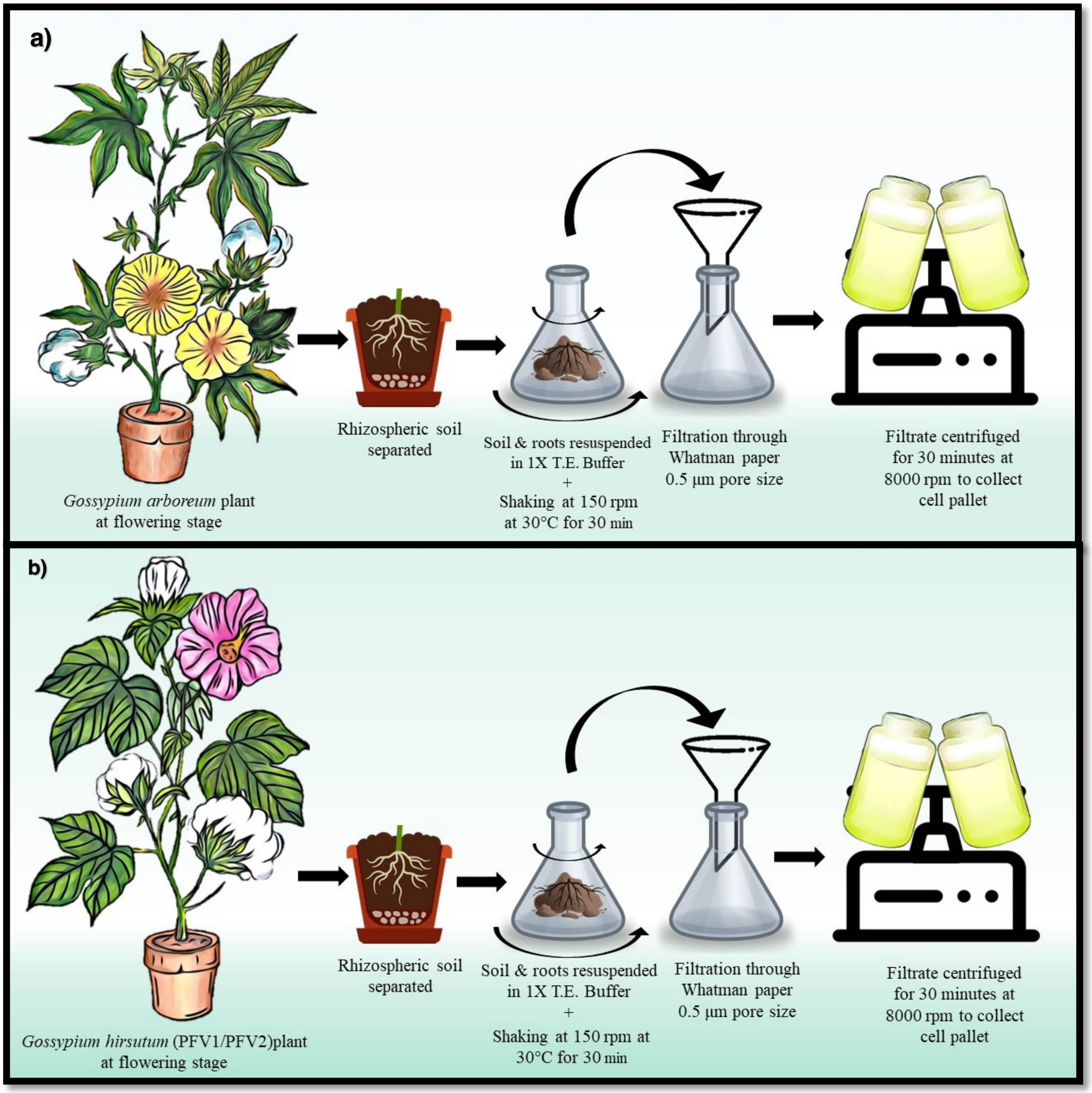

Supplementary Figure 18. **Rhizospheric Microbial Fraction Preparation**. **a)** Rhi.RMF preparation from *Gossypium arboreum* FDH228 plant rhizospheric soil. **b)** Rhi.pRMF and Rhi.SMF preparation from *Gossypium hirsutum* PFV1 and PFV2 plant rhizospheric soil, respectively.

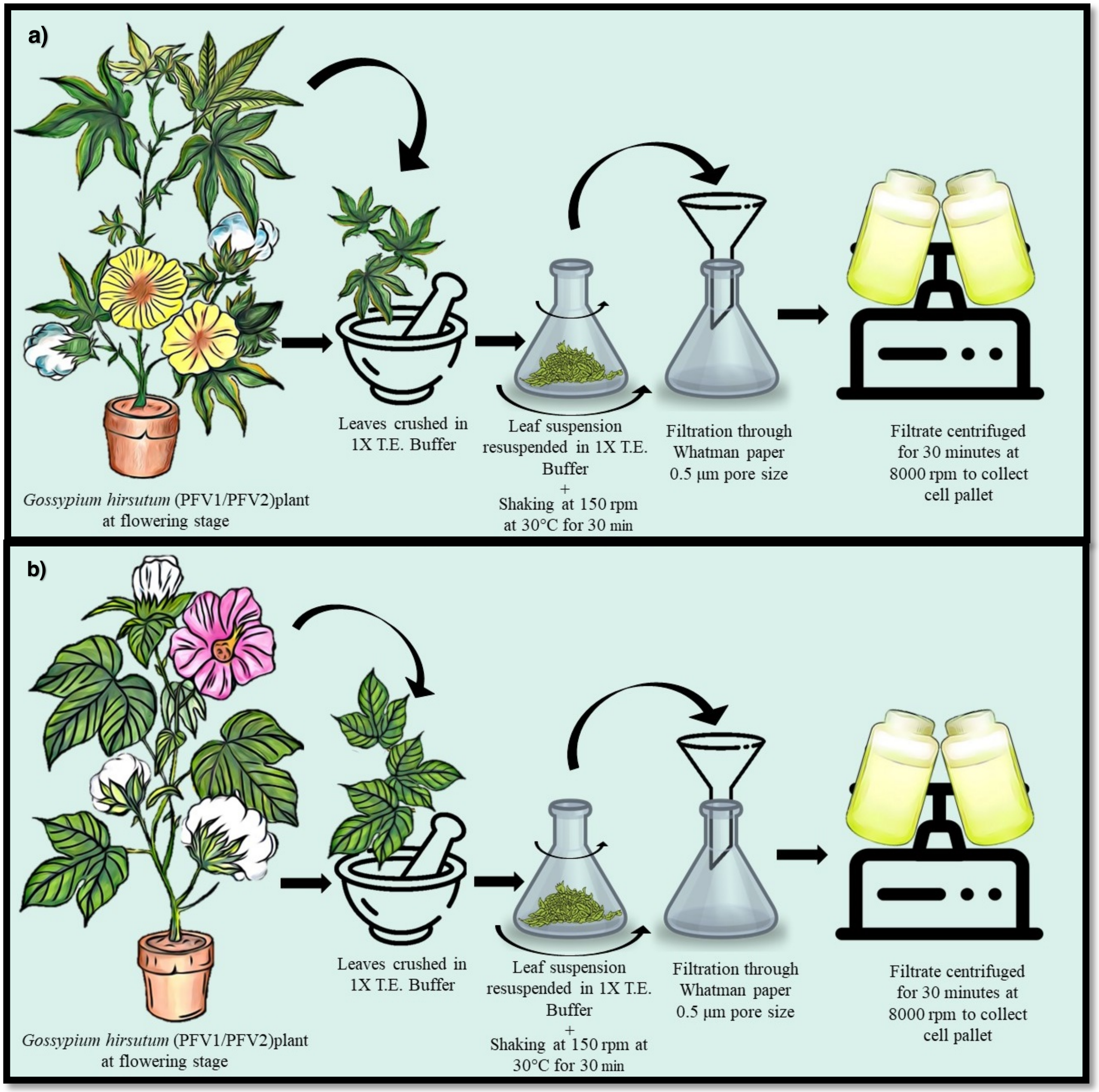

Supplementary Figure 19. **Phyllospheric Microbial Fraction Preparation**. **a)** Phy.RMF preparation from *Gossypium arboreum* FDH228 plant phyllosphere. **b)** Phy.pRMF and Phy.SMF preparation from *Gossypium hirsutum* PFV1 and PFV2 plant phyllosphere, respectively.

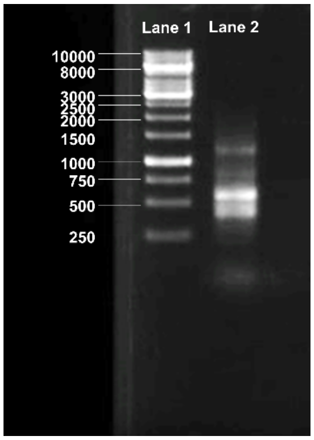

Supplementary Figure 20. β-satellite amplification for identification of CLCuV infection in *G. arboreum* and *G. hirsutum* test plants Lane 1: 1 kb Gene Ruler, Lane 2: β-satellite complex of Begomovirus

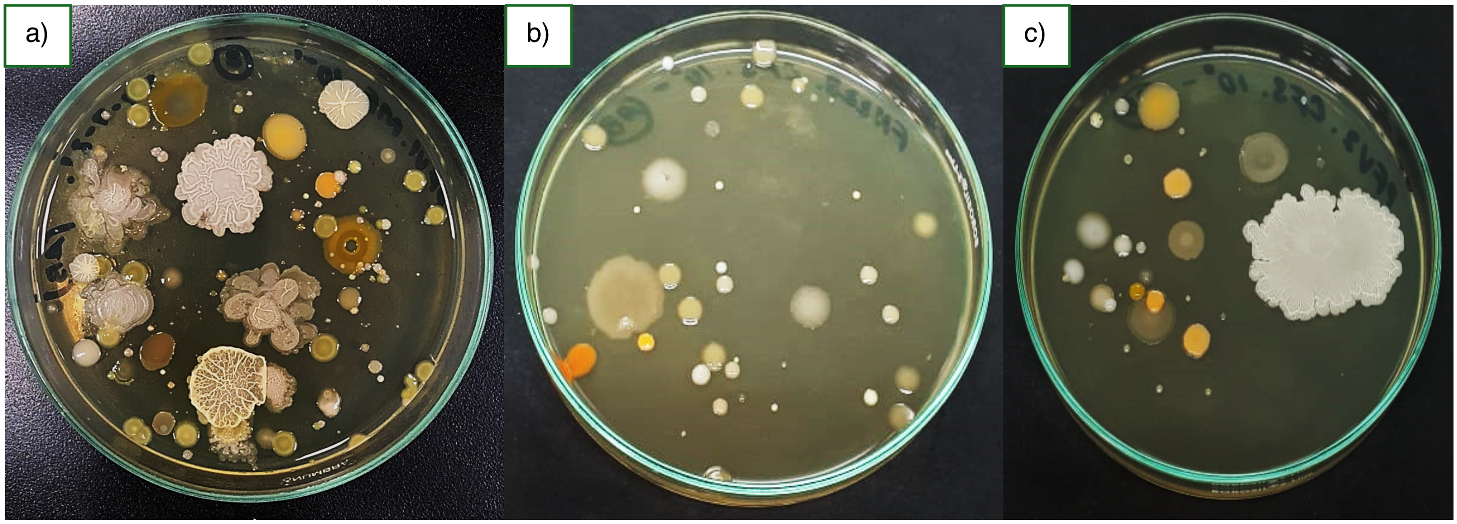

Supplementary Figure 21. Microbial Fraction plated onto half-strength Tryptic Soy Agar. **a)** 100 µL RMF (Microbial Fraction extracted from FDH228 rhizosphere). **b)** 100 µL pRMF (Microbial Fraction extracted from PFV1 rhizosphere. **c)** 100 µL SMF (Microbial Fraction extracted from PFV2 rhizosphere.

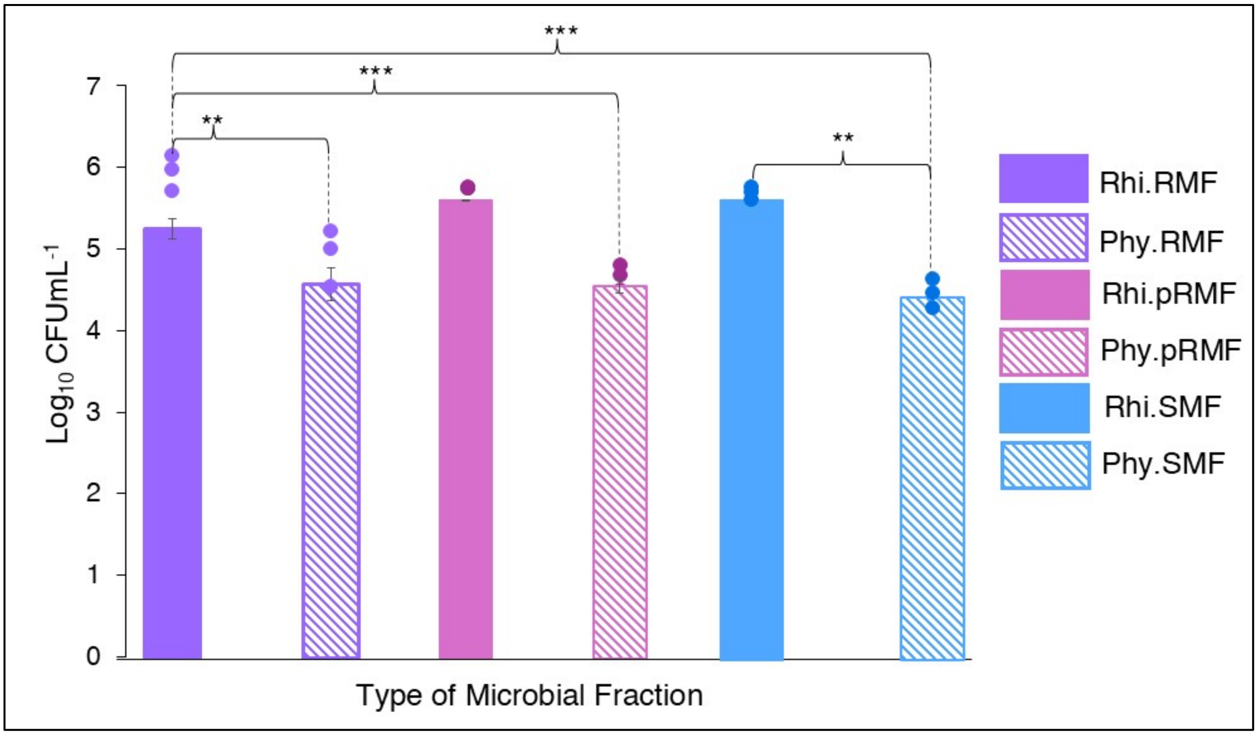

Supplementary Figure 22. Culture-based quantification of bacterial cell load in Microbial Fraction samples in CFUmL^{-}

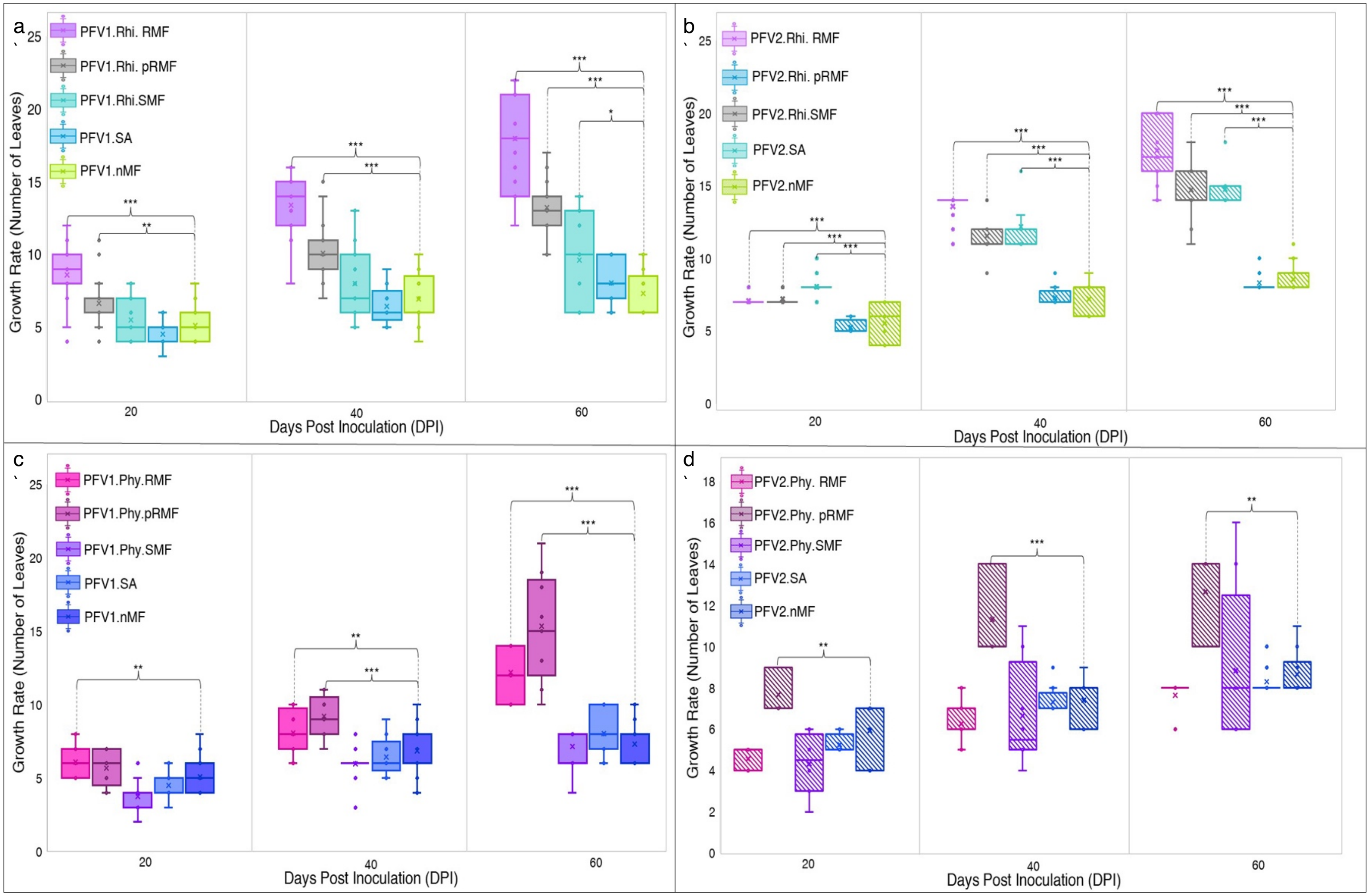

Supplementary Figure 23. Plant growth profiles post MF transplantation. a) Growth profiles of rhizosphere transplant groups for *G. hirsutum* PFV1 against SA and nMF, **b)** Growth profiles of rhizosphere transplant groups for *G. hirsutum* PFV2 against SA and nMF **c)** Growth profiles of phyllosphere transplant groups for *G. hirsutum* PFV1 against SA and nMF **d)** Growth profiles of phyllosphere transplant groups for *G. hirsutum* PFV2 against SA and nMF.

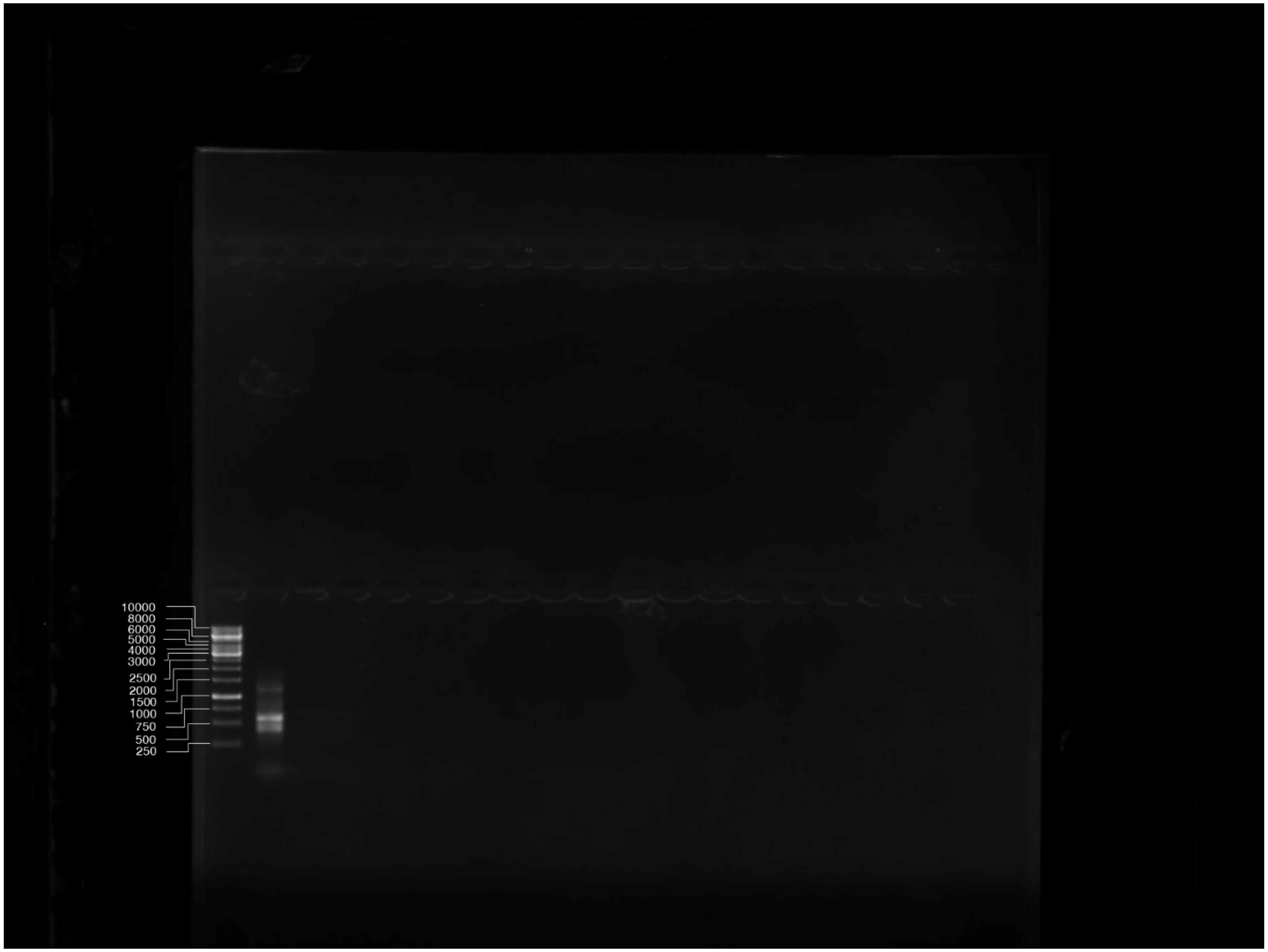

Supplementary Figure 24. Uncropped and unedited gel image for Supplementary Figure 20.

Supplementary References

1. Arshad, M. et al. Registration of ‘CIM-496’Cotton. *J. Plant Regist*. **3**, 231-235 (2009).
2. ahman, M., & Zafar, Y. Registration of NIBGE-115 cotton. *J. Plant Regist*. **1**, 51-52 (2007).
3. Arshad, M. et al. Registration of ‘CIM-496’Cotton. *J. Plant Regist*. **3**, 231-235 (2009).
4. Rahman, M. et al. Cotton germplasm of Pakistan world cotton germplasm resources, in World Cotton Germplasm Resources, ed. I. Y. Abdurakhmonov (New Delhi: Intex). 138–166 (2014).
5. Rahman, M., Hussain, D., Malik, T.A., Zafar, Y. Genetics of resistance to cotton leaf curl disease in Gossypium hirsutum. *Plant Pathol*. **54**, 764-772 (2005).
6. Ngou, B.P.M., Ahn, H.K., Ding, P., Jones, J.D. Mutual potentiation of plant immunity by cell-surface and intracellular receptors. *Nature*. **592**, 110-115 (2021).
7. Naqvi, R.Z. Transcriptomics reveals multiple resistance mechanisms against cotton leaf curl disease in a naturally immune cotton species, Gossypium arboreum. *Sci.rep*. **7**, 1-15 (2017).
8. Trivedi, P., Leach, J.E., Tringe, S.G., Sa, T., Singh, B.K. Author Correction: Plant–microbiome interactions: from community assembly to plant health. *Nat.rev.microbiol*. **19**, 72-72 (2021).
9. Liu, H. et al. Evidence for the plant recruitment of beneficial microbes to suppress soil-borne pathogens. *New.Phytol.* **229**, 2873-2885 (2021).
10. Schulz-Bohm, K. et al. Calling from distance: attraction of soil bacteria by plant root volatiles. *ISME.J*. **12**, 1252-1262 (2018).
11. Gu, S. et al. Competition for iron drives phytopathogen control by natural rhizosphere microbiomes. *Nat.Microbiol*. **5**, 1002-1010 (2020).
12. Berendsen, R.L. et al. Disease-induced assemblage of a plant-beneficial bacterial consortium. *ISME.J.* **12**, 1496-1507 (2018).
13. Huang, L. et al. Genome-Wide Analysis of the GW2-Like Genes in Gossypium and Functional Characterization of the Seed Size Effect of GhGW2-2D. *Front.plant.sci*. **13**, (2022).

14. Li, R., & Erpelding, J.E. Genetic diversity analysis of Gossypium arboreum germplasm accessions using genotyping-by-sequencing. *Genetica*. **144**, 535–545 (2016).
15. Cordovez, V., Dini-Andreote, F., Carrión, V.J., Raaijmakers, J.M. Ecology and evolution of plant microbiomes. *Annu.Rev.Microbiol.* **73**, 69-88 (2019).
16. Gong, T., & Xin, X.F. Phyllosphere microbiota: Community dynamics and its interaction with plant hosts. *J.Integr.Plant Biol*. **63**, 297-304 (2021).
17. Li, P.D. et al. The phyllosphere microbiome shifts toward combating melanose pathogen. *Microbiome*. **10**, 1-17 (2022).
18. Xiong, C. et al. Host selection shapes crop microbiome assembly and network complexity. *New.Phytol*. **229**, 1091-1104 (2021).
19. Laforest-Lapointe, I., Messier, C., Kembel, S.W. Host species identity, site and time drive temperate tree phyllosphere bacterial community structure. *Microbiome*. **4**, 1-10 (2016).
20. de Vries, F.T. et al. Soil bacterial networks are less stable under drought than fungal networks. *Nat.Commun*. **9**, 3033 (2018).
21. Fernández-González, A.J. et al. Linking belowground microbial network changes to different tolerance level towards Verticillium wilt of olive. *Microbiome*. **8**, 1-19 (2020).
22. Yin, C. et al. Rhizosphere community selection reveals bacteria associated with reduced root disease. *Microbiome*. **9**, 1-18 (2021).
23. Carrión, V.J. et al. Pathogen-induced activation of disease-suppressive functions in the endophytic root microbiome. *Science* https://doi.org/10.1126/science.aaw9285 (2019).
24. Schulz-Bohm, K. et al. Calling from distance: attraction of soil bacteria by plant root volatiles. *ISME.J*. **12**, 1252-1262 (2018).
25. Yuan, J. et al. Root exudates drive the soil-borne legacy of aboveground pathogen infection. *Microbiome* https://doi.org/10.1186/s40168-018-0537-x (2018).
26. Berg, M., & Koskella, B. Nutrient-and dose-dependent microbiome-mediated protection against a plant pathogen. *Curr.Biol*. **28**, 2487-2492 (2018).

27. Vannier, N., Agler, M., Hacquard, S. Microbiota-mediated disease resistance in plants. *PLoS.Pathog.* **15**, 1007740 (2019).
28. Hacquard, S., Spaepen, S., Garrido-Oter, R., Schulze-Lefert, P. Interplay between innate immunity and the plant microbiota. *Annu.Rev.Phytopathol*. **55**, 565-589 (2017).
