# Supplementary material for: Microbiota transplantation for cotton leaf curl disease suppression—core microbiome and transcriptome dynamics
Source: Commun Biol. 2025 Mar 6;8:380. doi: 10.1038/s42003-025-07812-7 (PMC11885576; doi:10.1038/s42003-025-07812-7)
Supplement: Supplementary file 2 — Description of Additional Supplementary Files [file 42003_2025_7812_MOESM2_ESM.docx]

Description of Additional Supplementary Files

**File name:** Supplementary Data 1

**Description:** RNA-Seq Metadata and Results Summary.

**File name:** Supplementary Data 2

**Description:** Bacterial quantification raw data and statistical analysis.

**File name:** Supplementary Data 3

**Description:** Disease severity assays raw data and statistical analysis.

**File name:** Supplementary Data 4

**Description:** Metadata File.
